# Cost-effectiveness of maternal pertussis immunization: Implications of a dynamic transmission model for low- and middle-income countries

**DOI:** 10.1016/j.vaccine.2020.09.012

**Published:** 2021-01-03

**Authors:** Sun-Young Kim, Kyung-Duk Min, Sung-mok Jung, Louise B. Russell, Cristiana Toscano, Ruth Minamisava, Ana Lucia S. Andrade, Colin Sanderson, Anushua Sinha

**Affiliations:** a Department of Public Health Sciences, Graduate School of Public Health, Seoul National University, Seoul, Republic of Korea; b Institute of Health and Environment, Graduate School of Public Health, Seoul National University, Seoul, Republic of Korea; c Department of Medical Ethics and Health Policy, Perelman School of Medicine, University of Pennsylvania, Philadelphia, PA, United States; d Institute of Tropical Pathology and Public Health, Federal University of Goiás, Goiânia, Goiás, Brazil; e School of Nursing, Federal University of Goiás, Goiânia, Goiás, Brazil; f Institute of Tropical Pathology and Public Health, Federal University of Goiás, Goiânia, Goiás, Brazil; g London School of Hygiene and Tropical Medicine, Department of Health Services Research and Policy, London, United Kingdom; h Merck & Co, Rahway, NJ, United States

**Keywords:** Pertussis, Vaccine, Dynamic transmission model, Cost-effectiveness, Brazil, Low- and middle-income countries

## Abstract

**Objective:**

This study evaluates the cost-effectiveness of maternal acellular pertussis (aP) immunization in low- and middle-income countries using a dynamic transmission model.

**Methods:**

We developed a dynamic transmission model to simulate the impact of infant vaccination with whole-cell pertussis (wP) vaccine with and without maternal aP immunization. The model was calibrated to Brazilian surveillance data and then used to project health outcomes and costs under alternative strategies in Brazil, and, after adjusting model parameter values to reflect their conditions, in Nigeria and Bangladesh. The primary measure of cost-effectiveness is incremental cost (2014 USD) per disability-adjusted life-year (DALY).

**Results:**

The dynamic model shows that maternal aP immunization would be cost-effective in Brazil, a middle-income country, under the base-case assumptions, but would be very expensive at infant vaccination coverage in and above the threshold range necessary to eliminate the disease (90–95%). At 2007 infant coverage (DTP1 90%, DTP3 61% at 1 year of age), maternal immunization would cost < $4,000 per DALY averted. At high infant coverage, such as Brazil in 1996 (DTP1 94%, DTP3 74% at 1 year), cost/DALY increases to $1.27 million. When the model’s time horizon was extended from 2030 to 2100, cost/DALY increased under both infant coverage levels, but more steeply with high coverage. The results were moderately sensitive to discount rate, maternal vaccine price, and maternal aP coverage and were robust using the 100 best-fitting parameter sets. Scenarios representing low-income countries showed that maternal aP immunization could be cost-saving in countries with low infant coverage, such as Nigeria, but very expensive in countries, such as Bangladesh, with high infant coverage.

**Conclusion:**

A dynamic model, which captures the herd immunity benefits of pertussis vaccination, shows that, in low- and middle-income countries, maternal aP immunization is cost-effective when infant vaccination coverage is moderate, even cost-saving when it is low, but not cost-effective when coverage levels pass 90–95%.

## Highlights

• (84) Low- and middle-income countries (LMICs) have experienced a resurgence of pertussis.
• (74) Maternal aP immunization could prevent pertussis among very young infants.
• (84) A dynamic transmission model was used to evaluate maternal aP immunization in LMICs.
• (82) Maternal aP is cost-effective when infant vaccination coverage is moderate or low.
• (85) Maternal aP immunization is not cost-effective in LMICs with infant coverage 90-95%.

## 1 Introduction

Pertussis vaccine has been administered for decades worldwide, although heterogeneity in coverage and delays in achieving coverage have also been reported [1], [2]. Yet, despite reportedly high infant vaccination coverage, and in addition to its natural cyclical nature, with peaks in disease every 3 to 5 years, resurgent pertussis has been reported in many countries, especially among young infants. Single-dose maternal acellular pertussis (aP) immunization during pregnancy, which confers immunity on the infant through transplacental antibody transfer and reduces infants’ exposure to the pathogen by protecting their mothers, could efficiently prevent disease and death, particularly in infants in low- and middle-income countries (LMICs) [3]. Although the period of immunity conferred by transplacental antibody transfer is short, perhaps 6 months at most [4], maternal aP immunization has the potential to prevent pertussis in infants who are too young to be vaccinated. Based on the potential benefits, the World Health Organization recommended that countries consider implementation of maternal pertussis immunization [5].

As of 2018, sixteen countries, have implemented maternal aP immunization. The majority is high-income, and five middle-income countries (Argentina, Brazil, Colombia, El Salvador, and Mexico) have incorporated maternal aP immunization into their routine vaccination schedules [6]. No low-income countries have done so, although these countries’ existing maternal immunization programs, which already provide tetanus toxoid, offer a platform with untapped potential to provide maternal aP immunization, although at additional cost.

The purpose of this study is to evaluate the cost-effectiveness of maternal aP immunization in LMICs, from the healthcare system perspective, using a dynamic transmission model. To build the model we drew on the rich data systems available in Brazil, a middle-income country. The goal is to provide insights into the cost-effectiveness profile of maternal immunization over time in LMICs in order to help policymakers identify best strategies for pertussis control. For comparability with other interventions for low- and middle-income settings, we have measured health outcomes in terms of disability-adjusted life years (DALYs).

## 2 Methods

To capture the dynamics of pertussis epidemiology, we developed an age-stratified dynamic transmission model and fitted the model to reported cases of pertussis in Brazil for the period 1999–2016. Using the model, we projected health and cost outcomes of two strategies: infant whole-cell pertussis (wP) vaccination only, and infant wP vaccination plus maternal aP immunization, from 2017 to 2030. Based on the model outcomes, we estimated the cost-effectiveness of adding maternal aP immunization to infant wP vaccination in terms of incremental cost per DALY averted. In developing the dynamic transmission model, we followed the recommendations of the ISPOR-SMDM Modeling Good Research Practices Task Force Working Group on dynamic transmission modeling [7].

The dynamic model was based on the rich data sources available for Brazil: surveillance, mortality and births, hospitalizations, and immunization. In Brazil, infant wP vaccine was first introduced into the routine immunization program in 1973 and is still used. A pertussis epidemic began in 2011 and peaked in 2014 with more than 8,000 reported cases. Maternal aP immunization was introduced in late 2014, the peak year, as a response to the resurgent pertussis burden. However, annual maternal aP coverages since implementation (10.9% in 2014, 48.4% in 2015, and 33.8% in 2016) are low and thus the impact on pertussis epidemiology of adding maternal immunization to the existing infant vaccination schedule is not expected to be significant.

### 2.1 Model structure

To model the epidemiological states and transmission dynamics of pertussis in the entire Brazilian population, we developed a dynamic transmission model in the form of a deterministic compartmental model, including S(=Susceptible), I(=Infected/Infectious), and R(=Recovered) states. Given the uncertainty surrounding the epidemiology and natural history of pertussis, in order to identify a model structure that best fits the Brazilian data, four model variants were tested, each with different assumptions about immunity waning and repeat infections, building upon the examples of a recent pertussis dynamic modeling study [8] (see Appendix 1 for the model schematic of each variant and equations).

Model 1 (*SIR*): Assumes lifelong immunity for both vaccination and natural infection.
Model 2 (*SIRS*): Assumes immunity wanes and repeat infections occur with the same reporting rate as primary infections.
Model 3 (*SIRS*_2_*I*_2_): Assumes immunity wanes and repeat infections occur with a lower reporting rate and less severity.
Model 4 $\left(SIR_{2}^{B}I_{2}\right)$: Assumes immunity wanes, repeat infections are less likely to be reported, and susceptible individuals who have previously been infected or vaccinated may experience immunity boosting upon re-exposure.

The models included a V(=Vaccinated/Immune) state to simulate the impact of alternative pertussis vaccination strategies. The V state distinguished immunity due to routine infant or maternal pertussis vaccination and by dose up to three.

The dynamic model includes the entire population and is age-structured with 18 age groups (0–1 month, 2–3, 4–5, 6–8, 9–11, 12–23 months, 2–4 years, and 5–9, 10–14, 15–19, 20–29, 30–39, 40–49, 50–59, 60–69, 70–79 and 80 + years). The first year of age was subdivided into 5 groups to capture the impact of alternative pertussis vaccination schedules on pertussis outcomes among infants, the group with the highest pertussis disease burden. Within the dynamic model, each age group has a different contact rate with other age groups and age-group-specific transmission probabilities per contact. This allows the model to reflect the transmission dynamics of pertussis infection in the entire population, calculating the force of infection (the rate at which susceptible individuals become infected) as a function of the number of individuals infected over time.

The fact that pertussis cases (especially outpatient cases) are significantly under-reported has to be taken into consideration and the force of infection adjusted for under-reporting. Therefore, the model explicitly incorporated a reporting rate parameter.

Initial conditions of each compartment in each variant of the model were estimated based on the reporting rate, historical data on reported pertussis cases, vaccination coverage rates, and mortality and births. Appendix 2 provides more details about the estimation process for the initial conditions.

### 2.2 Model inputs

Table 1 presents the key model inputs. Pertussis disease burden data were obtained from various sources. Pertussis deaths were obtained from the Brazilian mortality system (*Sistema de Informação sobre Mortalidade,* SIM). We assumed that all deaths are reported (no under-reporting) and thus we used the data as reported by the mortality system. Inpatient pertussis cases hospitalized in the public health system were obtained from the public health system (SUS) hospitalization information system (*Sistema de Informações Hospitalares,* SIH-SUS). Like the deaths, we assumed that, due to their severity, all hospitalized pertussis cases are reported but we adjusted the number of hospitalized cases reported to SIH-SUS in each Brazilian state by the state’s SUS coverage rate, on the assumption that the reporting rate in the private system was the same. We used pertussis cases reported to the National Notifiable Diseases Surveillance Information System (*Sistema de Informação de Agravos de Notificação,* SINAN), taking confirmed cases (by laboratory, clinical, or epidemiological confirmation criteria) to represent the total number of symptomatic cases (outpatient cases, hospitalizations, and deaths). Our model assumed that approximately 50% of all pertussis infections would be symptomatic [9], [10].

**Table 1:** Key model inputs.

Symbol	Description	Value (Range)	Source
Natural history parameters			
**λ**	force of infection	c×q	Estimated within model
c	contact rate	See Appendix 3	Determined through calibration
q	transmission probability per contact	See Appendix 3	Determined through calibration
**γ**	recovery rate	1/21 days	Heymann (2004)
$\rho_{1}$	reporting rate among I_1_	0y: 8.3%, 1-9y: 7.1%, 10y+: 6.2%	Determined through calibration
$\rho_{2}$	reporting rate among I2 (multiplier relative to I1 reporting rate)	0.4	Determined through calibration
**μ*_p_***	pertussis specific mortality		Brazil data

Vaccine characteristics			
**ν*_C_*_1_**	wP vaccine coverage (1st dose)	90% (2007), 94% (1996)	Brazil data
**ν*_C_*_2_**	wP vaccine coverage (2nd dose)	79% (2007), 88% (1996)	Brazil data
**ν*_C_*_3_**	wP vaccine coverage (3rd dose)	61% (2007), 74% (1996)	Brazil data
$v_{M}$	maternal aP vaccine coverage	10.9% (2014) to 70% (2030)	Brazil data
**θ*_C_***	rate of wP vaccine failure	0.1 (0–0.15)	Magpantay (2016)
**η*_C_*_1_**	wP effectiveness (1st dose)	0.68 (95% CI: 0.456–0.811)	Juretzko (2002)
**η*_C_*_2_**	wP effectiveness (2nd dose)	0.92 (95% CI: 0.847–0.957)	Juretzko (2002)
**η*_C_*_3_**	wP effectiveness (3rd dose)	0.99 (95% CI: 0.989–1.000)	Juretzko (2002)
**θ*_M_***	rate of maternal vaccine failure	0.1 (0–0.50)	Bento (2016)
**η*_M_***	maternal aP effectiveness	0.91 (95% CI: 0.84–0.95)	Amirthalingam (2014)
***σ_v_***	duration of wP immunity	7.309 years (5–30 years)	Determined through calibration
***σ_M_***	duration of maternal aP immunity	3 months (2–6 months)	Van Rie (2005), Smallenburg (2014), Bento (2016)
***σ_R_***	duration of natural immunity	20 years (10–50 years)	Wirsing von König (1995), Choi (2016)

Costs (2014 USD)			
$C_{w}$	vaccine wastage rate	5% (0–15%)	Brazil data
$C_{mp}$	maternal vaccine price per dose	9.55 (5.00–15.00)	Brazil data
$C_{cp}$	child vaccine price per dose	2.71	Brazil data
$C_{cd}$	child vaccine delivery cost per dose	7.60 (SE: 0.50)	Brazil data
$C_{d}$	death-related cost per case	See Appendix 6	Brazil data
$C_{i}$	inpatient cost per case	See Appendix 6	Brazil data
$C_{o}$	outpatient cost per case	See Appendix 6	Brazil data

Contact rates between age groups were not available for Brazil and were estimated based on the Polish POLYMOD contact matrix [11], which, of all the POLYMOD matrices, most closely resembled Brazilian contact patterns. The rates were adjusted by the ratio of Brazilian to Polish household size. In addition, we made the matrix symmetrical (i.e., individuals from two different age groups have the same contact rates regardless of which group initiates the contact). We then aggregated the age groups in the matrix into three age groups (<1, 1-9y, and 10+) and defined a multiplier for each group to allow the age-group-specific rates to vary in the model-fitting process so that we could identify the best-fitting rates. Finally, each age group had a different transmission probability per contact (see Appendix 3 for more details about the contact rates and transmission probabilities per contact for age groups).

We initially used administrative data on infant vaccination coverage. Administrative data are generally known to overestimate true vaccine coverage, but are thought to be sufficiently accurate. The dynamic model showed that they were not, projecting that, at administrative coverage levels, pertussis would be eliminated in Brazil in six years, when instead incidence has increased [12]. For more accurate coverage data, we turned to two national vaccine coverage surveys conducted in Brazil in 2007 and 1996. The coverages reported for DTP1, DTP2, and DTP3 are 90%, 79%, and 61% for the 2007 survey data and 94%, 88%, and 74% for the 1996 survey (Appendix 4). Maternal TdaP administrative vaccination coverage was 10.9%, 48.4%, and 33.8%, respectively, for each year in 2014–2016. For the base-case analysis, we assumed that coverage would linearly increase to a maximum of 70% from 2017 to 2030.

For infant wP vaccine effectiveness, we used 68%, 92%, 99%, for doses 1, 2, and 3, based on the literature [13]. For maternal aP vaccine, we assumed 91% effectiveness for the first three months of infancy, also based on the literature (Appendix 5).

Appendix 6 describes the cost data used for our analysis, including vaccine prices and costs of hospital and outpatient care for pertussis. We assumed a 5% vaccine wastage rate for both infant and maternal vaccines. Maternal vaccine delivery cost was assumed to be zero, since, in Brazil, TdaP replaced the Td vaccine that was already being administered to pregnant women. The cost of routine delivery of infant wP vaccination was estimated to be $2.71/dose based on a primary national immunization costing study conducted in Brazil in 2013 [14].

### 2.3 Calibration and selection of the best-fitting model

Some of the model parameters are highly uncertain. We calibrated each variant to Brazilian data using the likelihood-based Akaike Information Criterion (AIC) to select the best-fitting model and determine the values of the highly uncertain parameters (see Appendix 7 for details). A total of 13 parameters were considered to be highly uncertain and were varied from distributions assigned to the parameters for calibration. The variables included: 6 age-group multipliers for the contact matrix, 3 age-group multipliers for the transmission probabilities per contact, 3 age-group reporting rates for outpatient cases, and the duration of wP induced immunity. The calibration target was reported (confirmed) pertussis cases. We used age-group-specific pertussis cases, but to keep the computational burden reasonable, we defined three aggregated age groups (<1, 1–9, and 10+ years of age).

The fourth variant of the model (Model 4) was determined to be excluded from the candidate models since it was realized during the exploratory parameter search phase for calibration that the model’s boosting related parameters are highly hypothetical and uncertain and have a potential to yield biologically less plausible outcomes (e.g., higher reinfection than primary infection under certain values of the boosting parameters). Thus, model fitting was conducted for the first three variants only. After fitting each model to the age-group-specific data over the period 1999–2016, we calculated AIC scores and selected the best fitting model based on the lowest AIC score.

### 2.4 Strategies

The best-fitting calibrated model is used to compare strategies over the period 2017–2030: (1) routine infant wP vaccination alone (administered at 2, 4, and 6 months); and (2) maternal aP immunization (using Tdap) plus routine infant wP vaccination. The latter strategy was based on the assumption that maternal aP immunization on top of moderate coverage would avert over 90% of infections and deaths.

### 2.5 Model outcomes

The model was used to project the numbers of pertussis cases (with and without adjustment for reporting rate), outpatient cases, hospitalizations, deaths, and DALYs due to pertussis over the time horizon 2017–2030. The model also estimated costs associated with outpatient visits, hospitalizations, and deaths due to pertussis. Both health and cost outcomes were discounted at 3%, and costs were expressed in 2014 US dollars.

### 2.6 Cost-effectiveness

For the base-case analysis, the model calculates incremental cost-effectiveness ratios (ICERs) for adding maternal aP immunization to infant wP vaccination. The ICER is expressed in terms of incremental cost per DALY averted. We followed the CHEERS (Consolidated Health Economic Evaluation Reporting Standards) statement for reporting the results of the cost-effectiveness analysis (see Appendix 8 for the CHEERS Checklist completed) [15].

### 2.7 Uncertainty analysis

We conducted one-way sensitivity analyses to examine the influence of key parameters on the cost-effectiveness results: level of infant vaccination coverage, maternal aP coverage, aP vaccine price for maternal immunization, and discount rate. The model time horizon was extended up to 2100 in sensitivity analysis, to explore the effects of alternative time periods over which pertussis vaccination would be provided. We checked the robustness of the results by repeating the simulations using the 100 best-fitting parameter sets with the lowest AIC scores.

### 2.8 Scenarios for low-income countries

The data needed to develop a dynamic transmission model are scarce or lacking in low-income countries. To explore the cost-effectiveness of maternal immunization in these settings we adapted the current model, using available data on vaccination coverage, costs, and infant mortality, for two low-income countries – Bangladesh and Nigeria. Both countries are currently vaccinating infants with an accelerated schedule (6, 10, 14 weeks). Bangladesh has high coverage [DTP3 coverage at 1 year: 91% from 2011 survey data] while coverage rates in Nigeria are low [DTP3 coverage at 1 year: 33% from 2008 survey data]. Recently, it has been proposed to switch from the accelerated schedule to a simplified schedule (6 weeks, 14 weeks, 9 months). Accordingly, we compared four strategies for the two countries: 1) infant vaccination with the accelerated schedule; 2) infant vaccination with the accelerated schedule plus maternal immunization; 3) infant vaccination with the simplified schedule; 4) infant vaccination with the simplified schedule plus maternal immunization.

For infant vaccination coverage, we used Demographic and Health Survey data from each country, the 2011 survey for Bangladesh and the 2008 survey for Nigeria. For maternal aP coverage we used the percentages of pregnant women with at least one prenatal visit (ANC1): 78.6% for Bangladesh in 2014 [16] and 65.8% for Nigeria in 2013 [17] and assumed that rate held for the projection period, 2017–2030. Lacking country data on costs, we adjusted all costs related to vaccination and treatment using the ratio of GDP per capita between each country and Brazil (a ratio of 0.09 for Bangladesh vs. Brazil and 0.27 for Nigeria vs. Brazil). We assumed that the pertussis death rate would be higher due to a higher case-fatality rate (CFR), which might be attributable to lower access to health services than in Brazil, and modified the pertussis death rate using the ratio of CFR of each country and Brazil (a ratio of 4.33 for Bangladesh vs. Brazil and 2.47 for Nigeria vs. Brazil) [18].

We conducted one-way sensitivity analysis for the low-income country scenarios, varying maternal aP coverage (ANC4, 31.2% for Bangladesh [16] and 51.1% for Nigeria [17]), maternal aP vaccine price ($0.50-$5), time horizon, and discount rate.

## 3 Results

### 3.1 Model fitting

**Fig. 1:**
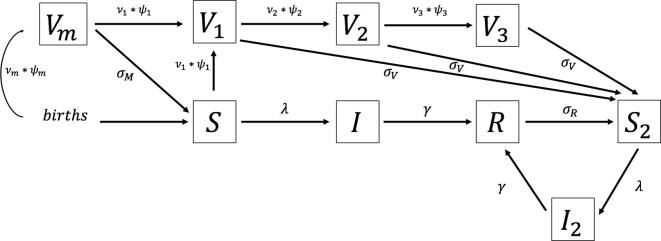
**Schematic of the best-fitting model (SIRS_2_I_2_).** The model assumes that both vaccine-induced and naturally-acquired immunity wane and repeat infection has a lower reporting rate than primary infection. Brief descriptions of each symbol in the schematic follow: S: Susceptible population. I: Infected and infectious population. R: Recovered and immune population from the infection. S_2_: Population with waning immunity from V or R compartments. I_2_: Population with secondary infection from S_2_. V_m_: Effectively immunized by maternal vaccination. V_1_: Effectively immunized by the 1st dose of child vaccination. V_2_: Effectively immunized by the 2nd dose of child vaccination. V_3_: Effectively immunized by the 3rd dose of child vaccination. ψ_i_: Proportion moving to protected compartments after vaccination (considering both primary vaccine failure and vaccine efficacy). v_i_: Proportion of individuals to be vaccinated (vaccine coverage). σ_V_: Waning rate of wP vaccine-induced immunity. σ_M_: Waning rate of aP vaccine-induced immunity. σ_R_: Waning rate of natural infection-induced immunity. λ: Force of infection. γ: Recovery rate.

**Fig. 2:**
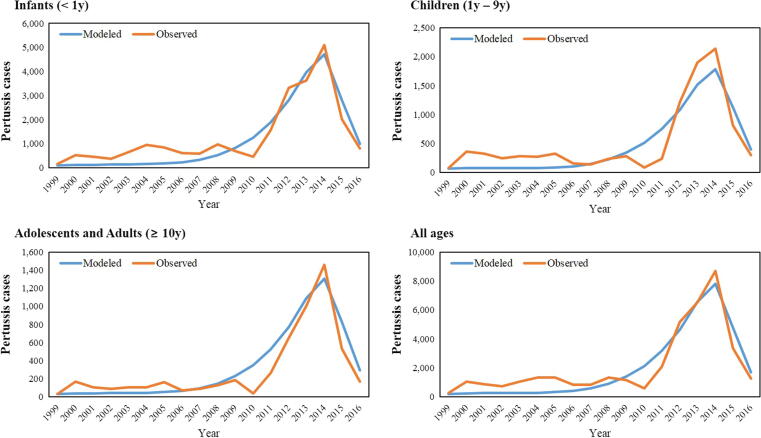
Best-fitting model (*SIRS*_2_*I*_2_): Observed (reported and confirmed) vs modeled pertussis cases by age group, 1999–2016.

Based on the AIC measure of goodness-of-fit, the third variant of the model, *SIRS*_2_*I*_2_, was selected as the best-fitting model, although the second variant, *SIRS*, was close and produced similar results (see Appendix 7 for the details of the calibration). Fig. 1 presents the schematic of the best-fitting model (*SIRS*_2_*I*_2_). Fig. 2 compares the observed data and projected outcomes using the *SIRS*_2_*I*_2_ model.

### 3.2 Base-case analysis: Brazil

Table 2 shows projected health and cost outcomes, 2017–2030, under two alternative infant vaccination coverage levels in Brazil (base-case rates based on 2007 survey data and higher coverage rates based on 1996 survey data). A majority of pertussis cases would be outpatient cases and the death rate would be very low. Yet, as is typically the case, most of the DALY burdens are attributable to pertussis deaths. Assuming infant vaccination coverage at the level of 2007, total program costs, 2017–2030, for infant vaccination would be $841 million while costs for maternal aP immunization would be $156 million (see Table 2).

**Table 2:** Brazil: Costs, health outcomes, and cost-effectiveness, infant vaccination and infant plus maternal vaccination, by infant vaccination coverage.

Model outcomes			Infant vaccination coverage			
			Base Case (2007 survey)		Higher (1996 survey)	
			Infant only	Infant + Maternal	Infant only	Infant + Maternal
Costs (2014 USD)	Treatment	outpatient	14,440,733	168,719	152,562	77,261
		inpatient	70,860,707	789,176	707,118	343,771
		death	769,635	8,855	6,909	3,491
		all	86,071,076	966,750	866,589	424,523
	Vaccination	dtp1	298,709,918	304,064,683	311,081,463	311,106,401
		dtp2	283,603,047	288,987,747	302,658,962	302,326,162
		dtp3	258,355,868	263,033,627	286,431,095	286,072,388
		wP vaccine + delivery	840,668,833	856,086,057	900,171,520	899,504,951
		aP (vaccine cost only)	–	156,304,969	–	156,304,969
		all	840,668,833	1,012,391,026	900,171,520	1,055,809,920
	Total cost		926,739,910	1,013,357,776	901,038,109	1,056,234,443

Health outcomes	Cases	outpatient	1,135,030	10,367	9,354	4,687
		inpatient	171,006	1,522	1,311	644
		death	901	8	6	3
	DALYs	YLD_outpt	6,060	71	64	33
		YLD_inpt	908	10	9	4
		YLD_death	5	0	0	0
		YLL_death	20,464	238	177	91
		DALY_total	27,437	319	251	128

Cost-effectiveness		DALYs	27,437	319	251	128
		DALYs averted	–	27,118	–	123
		Costs	926,739,910	1,013,357,776	901,038,109	1,056,234,443
		Incremental costs	–	86,617,866	–	155,196,334
		Cost/DALY	–	3,194	–	1,265,552*

* This figure may look different from that calculated using the rounded figures for DALYs averted (i.e., 123 DALYs), and incremental costs ($155,196,334) due to rounding error.

**Table 3:** Nigeria and Bangladesh: Cost-effectiveness of maternal aP immunization.

Country	Vaccination strategy*	DALYs	DALYs averted	Cost (2014 USD)	Incremental cost	Cost/DALY (2014 USD)
Nigeria (maternal aP coverage = 65.8%; see methods)	proposed simplified infant schedule	491,214		341,713,566	–	–
	current accelerated infant schedule	479,519	11,695	336,084,494	−5,629,072	See below
	simplified infant schedule + maternal aP	22,531	456,988	229,159,278	−106,925,216	See below
	accelerated infant schedule + maternal aP	17,021	5,509	223,893,852	−5,265,426	Cost-saving (i.e., averts more DALYs than the alternatives and saves money)

Bangladesh (maternal aP coverage = 78.6%; see methods)	proposed simplified infant schedule	699		80,653,133	–	–
	current accelerated infant schedule	627	73	81,685,180	1,032,047	14,151
	simplified infant schedule + maternal aP	252	374	101,905,88	20,220,707	54,031
	accelerated infant schedule + maternal aP	243	9	102,948,460	1,042,573	116,656

* Strategies for each country are listed in order of DALYs averted, with the strategy that averts the fewest DALYs at the top, the strategy that averts the most DALYs at the bottom.

The bottom panel of Table 2 shows the cost-effectiveness of maternal aP immunization for two infant coverage levels. With moderate coverage, based on the 2007 survey, maternal aP immunization would avert 27,118 DALYs at an incremental cost of about $86.6 million, costing $3,194 per DALY averted. With higher infant vaccination coverage, based on the 1996 survey, maternal aP immunization would avert only 123 DALYs at an additional cost of ~$155 million, and would cost $1.27 million per DALY averted.

### 3.3 Uncertainty analysis

**Fig. 3:**
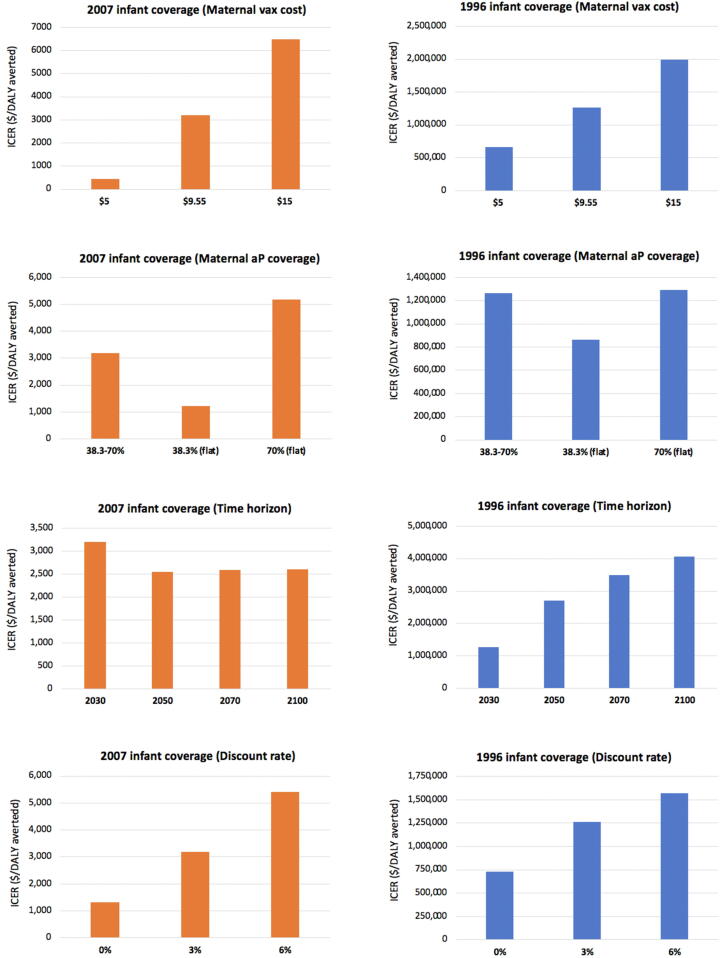
Brazil: Cost-effectiveness of maternal immunization under two alternative infant vaccination coverages. One-way sensitivity analysis.

Fig. 3 presents one-way sensitivity analysis results for Brazil. When maternal vaccine price is reduced to $5, about half the base-case value ($9.55), cost/DALY decreases to $448 and ~$658,000 under the moderate and high infant coverage levels, respectively. Cost/DALY increases modestly as the level of maternal coverage increases.

Cost/DALY is more sensitive to time horizon under high than moderate infant coverage. For instance, as the time horizon is extended from 2030 to 2100, cost/DALY under high infant coverage increases from ~$1.27 million to ~$4.07 million, while it changes very little under moderate infant coverage (Fig. 3).

**Fig. 4:**
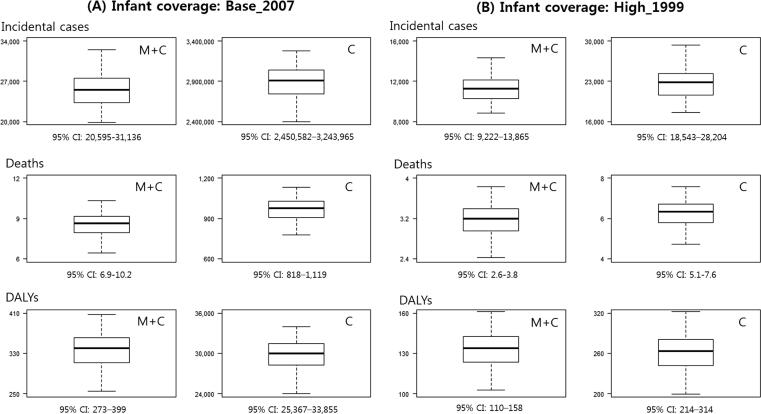
Uncertainty analysis: distributions of main health outcomes by infant coverage level, using the 100 best-fitting parameter sets. *M + C: maternal plus infant vaccination strategy; C: infant vaccination only strategy.

Simulations using the 100 best-fitting parameter sets (those with the lowest AIC scores) produced distributions for the main outcomes. Fig. 4 shows the distributions of the main health outcomes (incident cases, pertussis deaths, and DALYs), by strategy (infant plus maternal vs. infant only), and by level of infant vaccination coverage, along with 95% credible intervals (CIs). Overall, the plots show a low level of dispersion around the best-fit simulations.

**Fig. 5:**
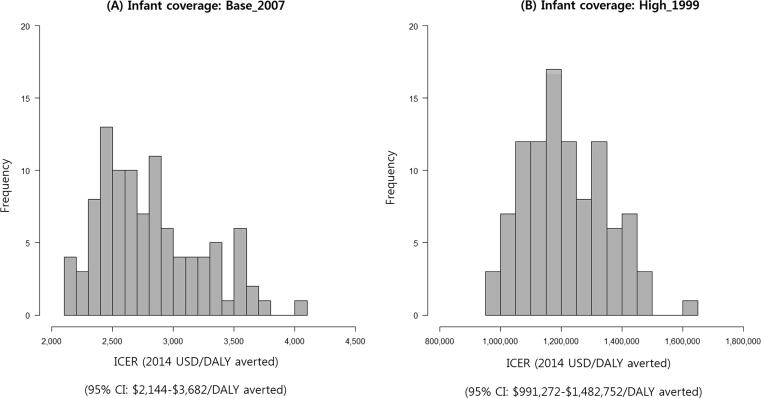
Uncertainty analysis: distributions of ICERs by infant coverage level, using the 100 best-fitting parameter sets.

Fig. 5 presents the distributions of cost/DALY by level of infant vaccination coverage, using the same 100 best-fitting parameter sets. The 95% CI of cost/DALY under the base-case (2007) infant coverage was $2,144-$3,682 and is skewed toward lower values. The distribution of cost/DALY under the high infant coverage (1996) had a more symmetric curve, with a 95% CI of $991,272-$1,482,752.

### 3.4 Nigeria and Bangladesh: Base case and sensitivity analyses

Table 3 provides the results of the scenario analyses developed for two example low-income countries, Nigeria and Bangladesh. Nigeria’s infant coverage level is much lower than that of Bangladesh.

**Table 4:** Nigeria and Bangladesh: Results by maternal aP vaccine price, time horizon, and maternal aP vaccine coverage.

Sensitivity	Country	Strategy	DALYs	DALYs averted	Cost (2014 USD)	Incremental cost	Cost/DALY (2014 USD)
**Maternal vaccine price**							
$0.5	Nigeria	Proposed simplified infant schedule	491,214		41,713,566		See below
		Current accelerated infant schedule	479,519	11,695	36,084,494	−5,629,072	See below
		Simplified infant schedule + maternal aP	22,531	456,988	185,831,203	−150,253,291	See below
		Accelerated infant schedule + maternal aP	17,021	5,509	180,565,778	−5,265,426	Cost-saving*
	Bangladesh	Proposed simplified infant schedule	699		80,653,133	–	–
		Current accelerated infant schedule	627	73	81,685,180	1,032,047	14,151
		Simplified infant schedule + maternal aP	252	374	92,948,007	11,262,828	30,095
		Accelerated infant schedule + maternal aP	243	9	93,990,580	1,042,573	116,656

$5	Nigeria	Proposed simplified infant schedule	491,214		341,713,566		See below
		Current accelerated infant schedule	479,519	11,695	336,084,494	−5,629,072	See below
		Simplified infant schedule + maternal aP	22,531	456,988	279,569,826	−56,514,668	See below
		Accelerated infant schedule + maternal aP	17,021	5,509	274,304,400	−5,265,426	Cost-saving*
	Bangladesh	Proposed simplified infant schedule	699		80,653,133	–	–
		Current accelerated infant schedule	627	73	81,685,180	1,032,047	14,151
		Simplified infant schedule + maternal aP	252	374	204,921,499	123,236,319	weakly dominated
		Accelerated infant schedule + maternal aP	243	9	205,964,072	1,042,573	323,643
**Time horizon**							
2017–2050	Nigeria	Proposed simplified infant schedule	914,617		628,769,113		See below
		Current accelerated infant schedule	894,333	20,284	618,832,793	−9,936,320	See below
		Simplified infant schedule + maternal aP	126,689	767,644	448,627,220	−170,205,572	See below
		Accelerated infant schedule + maternal aP	114,019	12,670	438,212,858	−10,414,363	Cost-saving*
	Bangladesh	Proposed simplified infant schedule	699		147,486,411	–	–
		Current accelerated infant schedule	627	73	149,002,790	1,516,379	20,791
		Simplified infant schedule + maternal aP	252	374	186,489,843	37,487,052	100,168
		Accelerated infant schedule + maternal aP	243	9	88,023,363	1,533,520	171,590

2017–2070	Nigeria	Proposed simplified infant schedule	1,145,182		786,834,301		See below
		Current accelerated infant schedule	1,120,201	24,982	774,515,816	−12,318,485	See below
		Simplified infant schedule + maternal aP	176,870	943,331	567,685,412	−206,830,404	See below
		Accelerated infant schedule + maternal aP	160,926	15,943	554,614,620	−13,070,792	Cost-saving*
	Bangladesh	Proposed simplified infant schedule	699		184,490,157	–	–
		Current accelerated infant schedule	627	73	186,274,900	1,784,743	24,471
		Simplified infant schedule + maternal aP	252	374	233,321,710	47,046,810	125,712
		Accelerated infant schedule + maternal aP	243	9	235,127,254	1,805,544	202,027

2017–2100	Nigeria	Proposed simplified infant schedule	1,312,754		901,914,144		See below
		Current accelerated infant schedule	1,284,354	28,400	887,859,930	−14,054,214	See below
		Simplified infant schedule + maternal aP	213,213	1,071,141	654,450,647	−233,409,283	See below
		Accelerated infant schedule + maternal aP	194,917	18,296	639,452,634	−14,998,013	Cost-saving*
	Bangladesh	Proposed simplified infant schedule	699		211,482,329	–	–
		Current accelerated infant schedule	627	73	213,462,827	1,980,499	27,155
		Simplified infant schedule + maternal aP	252	374	267,482,949	54,020,121	144,345
		Accelerated infant schedule + maternal aP	243	9	269,486,919	2,003,970	224,230
**Maternal aP coverage**							
Coverage based on ANC4 in each country	Nigeria (51.5%)	Proposed simplified infant schedule	491,214		341,713,566		See below
		Current accelerated infant schedule	479,519	11,695	336,084,494	−5,629,072	See below
		Simplified infant schedule + maternal aP	135,185	344,334	255,744,259	−80,340,235	See below
		Accelerated infant schedule + maternal aP	126,062	9,123	249,705,711	−6,038,548	Cost-saving*
	Bangladesh (31.2%)	Proposed simplified infant schedule	699		80,653,133	–	–
		Current accelerated infant schedule	627	73	81,685,180	1,032,047	14,151
		Simplified infant schedule + maternal aP	411	215	89,080,219	7,395,039	34,346
		Accelerated infant schedule + maternal aP	386	25	90,118,034	1,037,815	41,655

* Averts more DALYs and saves more cost than the alternatives.

As described, we evaluated four strategies for the two countries. For Nigeria, with its lower infant vaccination coverage, the accelerated schedule plus maternal aP immunization would be cost-saving and dominates all other strategies – that is, it saves more money and lives than any other strategy. For Bangladesh, with infant vaccination coverage higher even than Brazil’s 1996 coverage, the cost of adding maternal aP immunization to the accelerated infant schedule would be high, ~$117,000/DALY averted. These findings highlight the role of infant vaccination coverage in determining the cost-effectiveness of maternal aP immunization.

For Nigeria, the sensitivity analysis results were similar to the base case; for all parameter values examined, the accelerated schedule plus maternal aP immunization is the cost-saving and dominant strategy (Table 4). For Bangladesh, the sensitivity analysis results were also similar to the base-case: adding maternal immunization to either the accelerated or simplified infant schedule would be very expensive per DALY averted (Table 4).

## 4 Discussion

The dynamic model captures the herd immunity benefits of infant pertussis (wP) vaccination and suggests that, if policy makers are willing to spend GDP per capita to avert a DALY, maternal aP immunization would be cost-effective in Brazil at recent infant vaccination coverage rates, which have declined from the high rates achieved in the 1990s. When infant vaccination rates are in the 90–95% range or above, maternal aP vaccination does not represent good value, with incremental cost-effectiveness ratios exceeding $1 million per DALY averted.

These results contrast with an earlier cost-effectiveness analysis which found maternal aP vaccination to be cost-effective under a wide range of circumstances. That static state-transition model did not capture the herd protection benefits of infant coverage [19], because it did not consider the entire population but focused on infants only, and did not explicitly consider the fact that only a small proportion of pertussis diseases are reported through the surveillance system.

For Brazil, the results were robust in uncertainty analysis. When infant wP coverage was moderate, maternal aP vaccination remained cost-effective under a range of assumptions regarding time horizon. With infant coverage at a high level, longer time horizons made maternal aP an increasingly inefficient investment, with cost exceeding $4 million/DALY when the time horizon extended to 2100. Under high infant vaccination coverage, pertussis would be nearly eliminated by 2030, so that there is little additional health benefit from maternal immunization after 2030, but the costs of maternal immunization continue to be incurred, and thus cost/DALY averted rises.

If it is expected that the current moderate level of infant wP coverage will continue in Brazil, provision of maternal aP immunization would be a worthwhile intervention to prevent pertussis deaths among young infants. Given, however, that the cost-effectiveness of maternal aP immunization is sensitive to the level of infant vaccination coverage, our study highlights the importance of achieving and maintaining high levels of infant vaccination coverage. To support that achievement, it is necessary to have accurate information on the level of infant vaccination coverage [12].

Our study has important implications for low-income countries. Results for two scenarios designed to represent low-income countries show that maternal immunization could be cost-saving in countries with low infant vaccination coverage, but very expensive in countries with high infant vaccination coverage. Our findings also suggest that different infant vaccination schedules (the current accelerated vs. proposed simplified infant schedule) do not have much impact on this conclusion. These results should be interpreted with caution since the model used to produce them was fitted to data from a setting with different demography, socioeconomic status, and disease epidemiology. We note, however, that a separate dynamic model, fitted to data for three Brazilian states that vary widely in their sociodemographic characteristics, identified as best-fitting each state the same model structure identified here, suggesting that the nature of the disease and its transmission is similar across very different settings [20].

Our study has several limitations. First, due to the uncertainty about reporting rates and the quality of data from different sources, we had to make assumptions about some less well-known aspects of pertussis disease such as the proportion of cases that are symptomatic. Second, due to the computational burden of the calibration process, not all of the uncertain parameters (e.g., duration of maternal aP immunity and natural immunity) were varied through the modeling-fitting process. Third, due to the lack of Brazilian data on age-group-specific contact rates, we had to borrow a Polish contact matrix from the POLYMOD study conducted in Europe; we adjusted the contact matrix by applying the ratio of household size of the two countries. Fourth, due to a lack of incidence and vaccine coverage data for years before 1999, accurate calculations of the initial conditions were limited. Finally, the model was fitted to a country that uses wP vaccine for infants and may not apply to countries where infants receive aP vaccine.

Despite these limitations, our study suggests that a dynamic transmission model is a useful tool for projecting the potential health and cost outcomes of maternal aP immunization and generating evidence for policy formulation. For pathogens that are transmitted across age groups, and where interventions such as infant vaccination induce herd effects, maternal immunization models must consider transmission dynamics to produce accurate projections [21]. To our knowledge, our study is the first to evaluate the cost-effectiveness of maternal aP immunization in middle- or low-income countries using a dynamic model. The results suggest that maternal aP immunization has a role, but only when high levels of infant vaccination cannot be achieved.

## Data statement

The dataset used in this study are mostly provided in the Technical Appendix. Any additional data not shown in the Technical Appendix are available upon request from the corresponding author (Email: sykim22@snu.ac.kr).

## CRediT authorship contribution statement

**Sun-Young Kim:** Conceptualization, Formal analysis, Methodology, Writing - original draft, Writing - review & editing. **Kyung-Duk Min:** Data curation, Formal analysis, Visualization, Writing - review & editing. **Sung-mok Jung:** Data curation, Formal analysis, Writing - review & editing. **Louise B. Russell:** Conceptualization, Data curation, Validation, Writing - review & editing. **Cristiana Toscano:** Conceptualization, Data curation, Writing - review & editing. **Ruth Minamisava:** Data curation, Writing - review & editing. **Ana Lucia S. Andrade:** Data curation, Writing - review & editing. **Colin Sanderson:** Data curation, Writing - review & editing. **Anushua Sinha:** Conceptualization, Validation, Writing - review & editing.

## Declaration of Competing Interest

The authors declare that they have no known competing financial interests or personal relationships that could have appeared to influence the work reported in this paper. [Dr. Sinha is currently an employee at Merck & Co and has stock holdings in that company. She was at Rutgers New Jersey Medical School when this work was conducted. Merck & Co. played no role in study design, collection, analysis, interpretation of data, writing of the report, or in the decision to submit the paper for publication. They accept no responsibility for the contents. Pej Rohani served as a paid consultant on structuring and calibrating the dynamic model.]
