# Supplementary material for: Cost-effectiveness of maternal pertussis immunization: Implications of a dynamic transmission model for low- and middle-income countries
Source: Vaccine. 2021 Jan 3;39(1):147–57. doi: 10.1016/j.vaccine.2020.09.012 (PMC7735375; doi:10.1016/j.vaccine.2020.09.012)

**Cost-effectiveness of maternal pertussis immunization in Brazil: Use of a dynamic transmission model and implications for low- and middle-income countries**

**Technical Appendices**

**List of Appendices**

1. Model schematic and equations for the four variants of the dynamic transmission model
2. Estimation of initial conditions
3. Contact matrix and transmission probability per contact
4. Infant vaccine coverage
5. Vaccine efficacy
6. Cost data
7. Calibration methods
8. CHEERS Checklist

**Appendix 1. Model schematic and equations for the four variants of the dynamic transmission model**

Model 1: SIR

1. Schematics


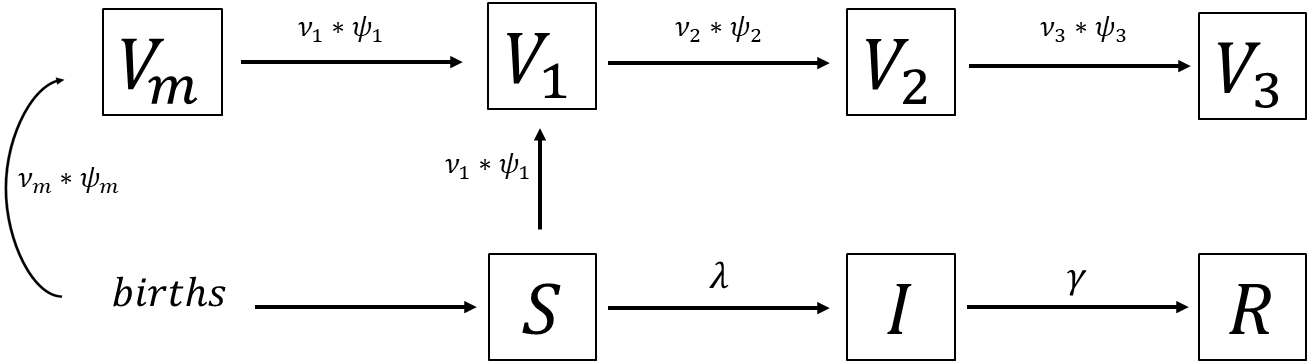


2. Key Assumptions

① The SIR model is composed of Susceptible (S), Infected (I), and Recovered (R) compartments.

② The model assumes perfect vaccine induced and naturally acquired lifelong immunity.

3. Differential equations

$$\frac{dS}{dt}= B\left( t \right)\left( 1-v_{m}\psi_{m} \right)-(v_{1}\psi_{1}+\lambda+\mu(t))S$$

$$\frac{dI}{dt}=\lambda S-(\gamma+\mu\left( t \right))I$$

$$\frac{dR}{dt}= \gamma I-(\sigma_{R}+\mu\left( t \right))R$$

$$\frac{dV_{m}}{dt}= B(t)v_{m}\psi_{m}-(v_{1}\psi_{1}+\mu(t))V_{m}$$

$$\frac{dV_{1}}{dt}= v_{1}\psi_{1}S-(v_{2}\psi_{2}+\mu(t))V_{1}$$

$$\frac{dV_{2}}{dt}= v_{2}\psi_{2}V_{1}-(v_{3}\psi_{3}+\mu(t))V_{2}$$

$$\frac{dV_{3}}{dt}= v_{3}\psi_{3}V_{2}-\mu(t)V_{3}$$

*Where,*

*S: susceptible population*

*I: infected and infectious population*

*R: Recovered and immuned population from the infection*

*Vm: Effectively immunized by maternal vaccination*

*V1: Effectively immunized by 1st child vaccination*

*V2: Effectively immunized by 2nd child vaccination*

*V3: Effectively immunized by 3rd child vaccination*

$\psi_{i}$*: Proportion moving to protected compartments after vaccination (considering both effectiveness and failure*

$v_{i}$*: Proportion of subject to be vaccinated (vaccine coverage)*

$\lambda$*: Force of infection*

$\gamma$*: Recovery rate*

Model 2: SIRS

1. Schematics


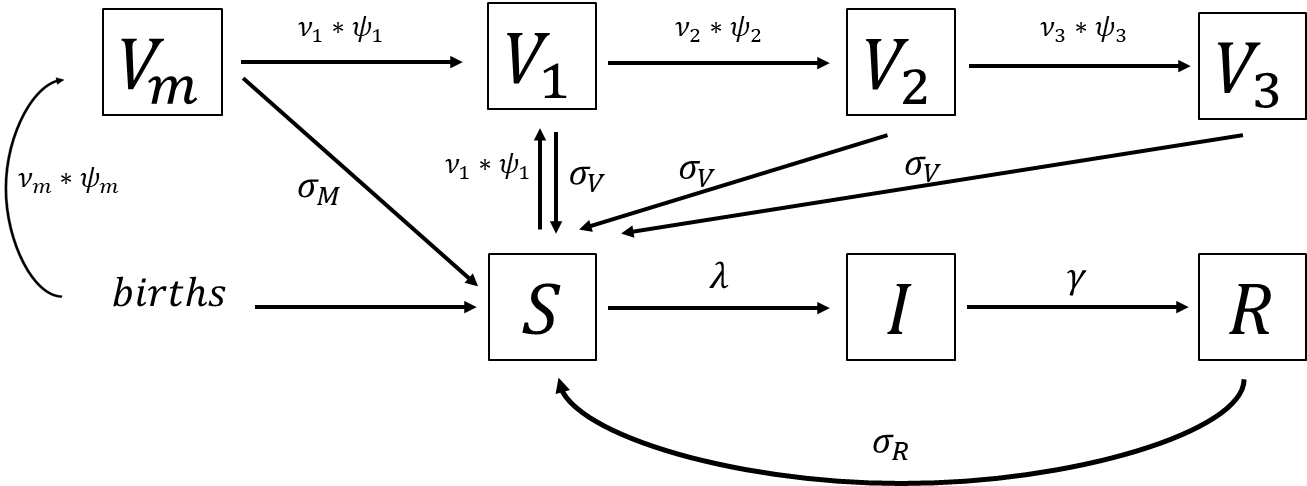


2. Key Assumptions

① Composed of Susceptible, Infected, Recovered, Susceptible2, Infected2 compartments

② Assumed vaccine induced and naturally acquired immunity wane

③ Assumed primary and secondary infection were reported with same probability

3. Differential equations

$$\frac{dS}{dt}= B\left( t \right)\left( 1-v_{m}\psi_{m} \right)-\left( v_{1}\psi_{1}+\lambda+\mu\left( t \right) \right)S+V_{m}\sigma_{M}+\left( V_{1}+V_{2}+V_{3} \right)\sigma_{V}+R\sigma_{R}$$

$$\frac{dI}{dt}=\lambda S-(\gamma+\mu\left( t \right))I$$

$$\frac{dR}{dt}= \gamma I-(\sigma_{R}+\mu\left( t \right))R$$

$$\frac{dV_{m}}{dt}= B(t)v_{m}\psi_{m}-(v_{1}\psi_{1}+\sigma_{M}+\mu(t))V_{m}$$

$$\frac{dV_{1}}{dt}= v_{1}\psi_{1}S-(v_{2}\psi_{2}+\sigma_{V}+\mu(t))V_{1}$$

$$\frac{dV_{2}}{dt}= v_{2}\psi_{2}V_{1}-(v_{3}\psi_{3}+\sigma_{V}+\mu(t))V_{2}$$

$$\frac{dV_{3}}{dt}= v_{3}\psi_{3}V_{2}-(\sigma_{V}+\mu(t))V_{3}$$

*Where,*

*S: susceptible population*

*I: infected and infectious population*

*R: Recovered and immuned population from the infection*

*Vm: Effectively immunized by maternal vaccination*

*V1: Effectively immunized by 1st child vaccination*

*V2: Effectively immunized by 2nd child vaccination*

*V3: Effectively immunized by 3rd child vaccination*

$\psi_{i}$*: Proportion moving to protected compartments after vaccination (considering both effectiveness and failure*

$v_{i}$*: Proportion of subject to be vaccinated (vaccine coverage)*

$\sigma_{V}$*: Waning rate of wP vaccine-induced immunity*

*σ_M_: Waning rate of aP vaccine-induced immunity*

$\sigma_{R}$*: Waning rate of natural infection-induced immunity*

$\lambda$*: Force of infection*

$\gamma$*: Recovery rate*

Model 3: SIRS_2_I_2_

1. Schematics


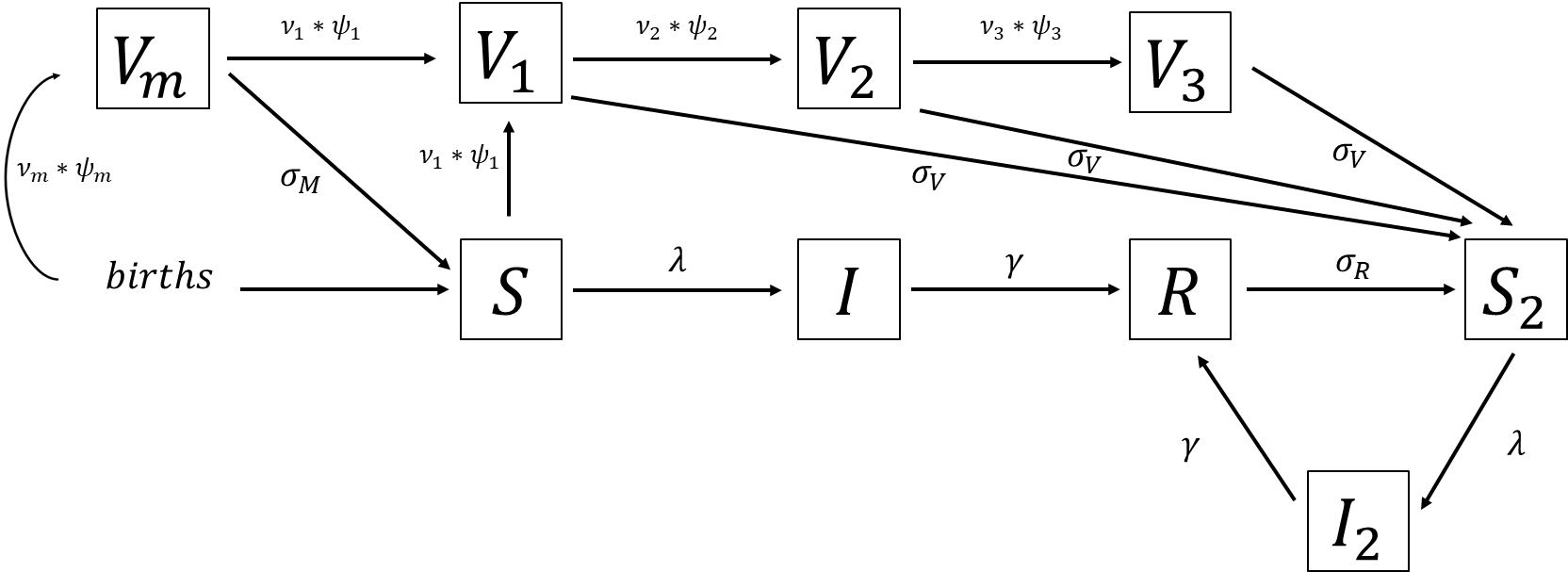


2. Key Assumptions

- 1. Composed of Susceptible, Infected, Recovered, Susceptible2, Infected2 compartments
  2. Assumed vaccine induced and naturally acquired immunity wane
  3. Assumed repeat infections have lower reporting probabilities than primary infection

3. Differential equations

$$\frac{dS}{dt}= B\left( t \right)\left( 1-v_{m}\psi_{m} \right)+ V_{m}\sigma_{M}-(v_{1}\psi_{1}+\lambda+\mu(t))S$$

$$\frac{dI}{dt}=\lambda S-(\gamma+\mu\left( t \right))I$$

$$\frac{dR}{dt}= \gamma(I+I_{2})-(\sigma_{R}+\mu\left( t \right))R$$

$$\frac{dS_{2}}{dt}= \sigma_{R}R+\sigma_{V}(V_{1}+ V_{2}+ V_{3})-\mu(t)S_{2}$$

$$\frac{dI_{2}}{dt}=\lambda S_{2}-(\gamma+\mu\left( t \right))I_{2}$$

$$\frac{dV_{m}}{dt}= B(t)v_{m}\psi_{m}-(v_{1}\psi_{1}+\sigma_{M}+\mu(t))V_{m}$$

$$\frac{dV_{1}}{dt}= v_{1}\psi_{1}S-(v_{2}\psi_{2}+\sigma_{V}+\mu(t))V_{1}$$

$$\frac{dV_{2}}{dt}= v_{2}\psi_{2}V_{1}-(v_{3}\psi_{3}+\sigma_{V}+\mu(t))V_{2}$$

$$\frac{dV_{3}}{dt}= v_{3}\psi_{3}V_{2}-(\sigma_{V}+\mu(t))V_{3}$$

*Where,*

*S: susceptible population*

*I: infected and infectious population*

*R: Recovered and immuned population from the infection*

*S2: Population with waning immunity from V compartments or R compartment*

*I2: Population with secondary infection from S2*

*Vm: Effectively immunized by maternal vaccination*

*V1: Effectively immunized by 1st child vaccination*

*V2: Effectively immunized by 2nd child vaccination*

*V3: Effectively immunized by 3rd child vaccination*

$\psi_{i}$*: Proportion moving to protected compartments after vaccination (considering both effectiveness and failure*

$v_{i}$*: Proportion of subject to be vaccinated (vaccine coverage)*

$\sigma_{V}$*: Waning rate of wP vaccine-induced immunity*

*σ_M_: Waning rate of aP vaccine-induced immunity*

$\sigma_{R}$*: Waning rate of natural infection-induced immunity*

$\lambda$*: Force of infection*

$\gamma$*: Recovery rate*

Model 4: SIRI^B^_2_S_2_

1. Schematics


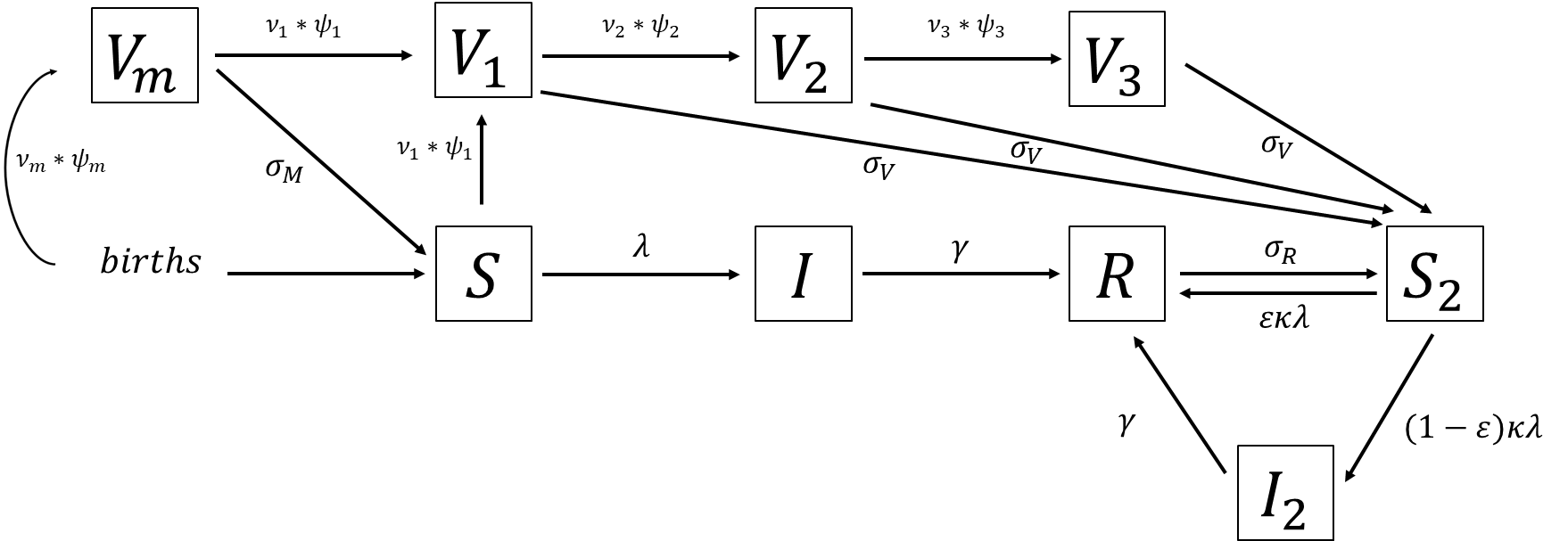


2. Key Assumptions

1. Composed of same compartments with SIRS_2_I_2_ model
2. Assumed vaccine induced and naturally acquired immunity wane
3. Assumed reduced reporting probability in secondary transmission
4. Assumed immunity of susceptible individuals who has previously been infected or vaccinated may be boosted upon re-exposure

**3. Differential equations**

$$\frac{dS}{dt}= B\left( t \right)\left( 1-v_{m}\psi_{m} \right)+V_{m}\sigma_{M}-(v_{1}\psi_{1}+\lambda+\mu(t))S$$

$$\frac{dI}{dt}=\lambda S-(\gamma+\mu\left( t \right))I$$

$$\frac{dR}{dt}= \gamma\left( I+I_{2} \right)-\left( \sigma_{R}+\mu\left( t \right) \right)R+ \epsilon\kappa\lambda S_{2}$$

$$\frac{dS_{2}}{dt}= \sigma_{R}R+\sigma_{V}(V_{1}+ V_{2}+ V_{3})-(\mu\left( t \right)+\kappa\lambda)S_{2}$$

$$\frac{dI_{2}}{dt}=(1-\epsilon)\kappa\lambda S_{2}-(\gamma+\mu\left( t \right))I_{2}$$

$$\frac{dV_{m}}{dt}= B(t)v_{m}\psi_{m}-(v_{1}\psi_{1}+\sigma_{M}+\mu(t))V_{m}$$

$$\frac{dV_{1}}{dt}= v_{1}\psi_{1}S-(v_{2}\psi_{2}+\sigma_{V}+\mu(t))V_{1}$$

$$\frac{dV_{2}}{dt}= v_{2}\psi_{2}V_{1}-(v_{3}\psi_{3}+\sigma_{V}+\mu(t))V_{2}$$

$$\frac{dV_{3}}{dt}= v_{3}\psi_{3}V_{2}-(\sigma_{V}+\mu(t))V_{3}$$

*Where,*

*S: susceptible population*

*I: infected and infectious population*

*R: Recovered and immuned population from the infection*

*S2: Population with waning immunity from V compartments or R compartment*

*I2: Population with secondary infection from S2*

*Vm: Effectively immunized by maternal vaccination*

*V1: Effectively immunized by 1st child vaccination*

*V2: Effectively immunized by 2nd child vaccination*

*V3: Effectively immunized by 3rd child vaccination*

$\psi_{i}$*: Proportion moving to protected compartments after vaccination (considering both effectiveness and failure*

$v_{i}$*: Proportion of subject to be vaccinated (vaccine coverage)*

$\sigma_{V}$*: Waning rate of wP vaccine-induced immunity*

*σ_M_: Waning rate of aP vaccine-induced immunity*

$\sigma_{R}$*: Waning rate of natural infection-induced immunity*

$\lambda$*: Force of infection*

$\gamma$*: Recovery rate*

$\varepsilon$: *Immunity boosted upon reexposure*

$\kappa$: *Boosting coefficient*

**Appendix 2. Estimation of initial conditions**

Initial conditions (the distributions of population density across the model compartments at time 0) were estimated using the following estimation process


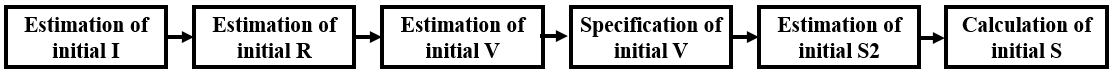


1. I compartment

Incidence cases of pertussis in Jan 1999, adjusted for under-reporting, was used as initial condition of I compartment.

2. I2 compartment

Initial condition of the I2 compartment was assumed to be zero in Model 3 (SIRS2I2).

3. R compartment

1. The number of monthly age-specific incidence cases from Jan 1918 to Dec 1998 was calculated by adjusting the number of age-specific notification cases for the reporting rate. To estimate age-specific notified cases from 1980 to 1988 and before 1980, we assumed that number of pertussis notification cases remained constant.
2. The number of incidence cases was further adjusted for the age-specific probability of surviving (derived from 1998 Brazilian life-table to adjust for probability of death over time).
3. As the initial condition of the R compartment is the cumulative effect of recovered individuals in each month, the initial condition was estimated by calculating diagonal sums of age-specific recovered individuals in each month from 1918 to 1998.

4. V compartment

For ages of 0-1 months, the number of individuals in the V compartment at time 0 (Jan 1999) were set at 0 based on the infant DTP vaccination schedule (2, 4, 6 months) in Brazil. For ages of 2-11 months, the initial conditions were assumed to be equal to the numbers of individuals vaccinated in Jan 1999.

5. S2 compartment

The initial condition for the S2 compartment was estimated considering immunity waning from the V compartment.

6. S compartment

The number of individuals in the S compartment at time 0 was calculated by subtracting the sum of the individuals in all other compartments from the population size for each age group.

**Appendix 3. Contact matrix and transmission probability per contact**

1. Use of the Polish POLYMOD contact matrix and adjustment

Since there are no empirical data for contact rates between age groups in Brazil, we adopted the Polish POLYMOD contact matrix to estimate the contact rates for the model. The Polish matrix was chosen based on the opinions of the Brazil experts that it seems to more close the contact pattern in general in Brazil than the matrices from other European POLYMOD countries.

The Polish matrix was modified by multiplying each element in it by 1.06, the ratio of Brazilian to Polish household size, since Brazil’s larger household size would increase transmission rates in all age groups. The data for household sizes in Poland were obtained from EUROSTAT data for 2005 to 2010 and UN-ECE data for 2000 and 2002. The data for Brazil were obtained from Census in 2000 and 2010.

Table A3-1. Modified POLYMOD (Polish) contact matrix adjusted for the ratio of household sizes between Brazil and Poland

|  | 00-04 | 05-09 | 10-14 | 15-19 | 20-24 | 25-29 | 30-34 | 35-39 | 40-44 | 45-49 | 50-54 | 55-59 | 60-64 | 65-69 | 70+ |
| --- | --- | --- | --- | --- | --- | --- | --- | --- | --- | --- | --- | --- | --- | --- | --- |
| 00-04 | 1.41 | 0.6 | 0.22 | 0.11 | 0.22 | 0.42 | 0.4 | 0.24 | 0.12 | 0.14 | 0.33 | 0.24 | 0.27 | 0.21 | 0.24 |
| 05-09 | 0.88 | 6.79 | 1.12 | 0.31 | 0.11 | 0.29 | 0.78 | 0.82 | 0.59 | 0.17 | 0.25 | 0.53 | 0.29 | 0.47 | 0.28 |
| 10-14 | 0.8 | 1.72 | 13.47 | 1.07 | 0.32 | 0.27 | 0.85 | 0.72 | 0.73 | 0.28 | 0.39 | 0.45 | 0.27 | 0.47 | 0.36 |
| 15-19 | 0.52 | 0.43 | 2.41 | 12.85 | 2.26 | 0.76 | 0.57 | 1.37 | 1.25 | 1.2 | 1 | 0.7 | 0.56 | 0.64 | 0.42 |
| 20-24 | 0.73 | 0.38 | 0.67 | 1.79 | 6.64 | 2.54 | 1.23 | 1.48 | 1.67 | 1.53 | 1.54 | 1.08 | 0.7 | 0.64 | 0.36 |
| 25-29 | 1.44 | 0.98 | 0.89 | 0.85 | 2.77 | 3.64 | 2.42 | 2.65 | 1.92 | 1.86 | 2.06 | 1.45 | 1.17 | 1.44 | 0.28 |
| 30-34 | 1.26 | 1.51 | 1.26 | 0.93 | 1.69 | 2.31 | 2.5 | 2.84 | 1.88 | 1.53 | 1.71 | 1.42 | 1.39 | 1.48 | 0.49 |
| 35-39 | 0.9 | 1.02 | 1.3 | 1.12 | 1.2 | 1.52 | 1.74 | 3.26 | 1.87 | 1.41 | 1.31 | 1.12 | 1.08 | 1.14 | 0.75 |
| 40-44 | 0.47 | 0.72 | 1.02 | 1.24 | 1.18 | 1.45 | 1.53 | 1.98 | 2.62 | 1.58 | 1.81 | 1.04 | 1.13 | 1.78 | 0.78 |
| 45-49 | 0.5 | 0.63 | 0.75 | 1.02 | 1.04 | 1.19 | 1.44 | 1.61 | 1.65 | 2.39 | 1.84 | 1.23 | 0.59 | 0.98 | 0.88 |
| 50-54 | 0.6 | 0.5 | 0.49 | 0.54 | 1.27 | 1.41 | 1.18 | 1.11 | 1.09 | 1.65 | 1.67 | 1.48 | 0.59 | 1.02 | 0.78 |
| 55-59 | 0.56 | 0.51 | 0.3 | 0.27 | 0.84 | 0.82 | 0.59 | 0.92 | 0.72 | 0.65 | 1 | 2.07 | 1.17 | 0.72 | 0.57 |
| 60-64 | 0.31 | 0.34 | 0.25 | 0.27 | 0.36 | 0.48 | 0.63 | 0.61 | 0.59 | 0.43 | 0.55 | 0.89 | 1.19 | 0.51 | 0.6 |
| 65-69 | 0.28 | 0.19 | 0.16 | 0.21 | 0.11 | 0.24 | 0.19 | 0.56 | 0.36 | 0.33 | 0.2 | 0.59 | 0.68 | 0.76 | 0.57 |
| 70+ | 0.3 | 0.38 | 0.4 | 0.24 | 0.32 | 0.42 | 0.32 | 0.75 | 0.58 | 0.71 | 0.75 | 0.82 | 0.68 | 0.85 | 1.3 |

1. Transformation of contact matrix for symmetry

The modified Polish POLYMOD contact matrix is not symmetric due to the nature of the data collection method through a survey. For internal consistency, we assumed that the contact rates between two age groups would be the same regardless which group initiates each contact. We then transformed the matrix to make it symmetric, using the method adopted from the Bento and Rohani’s study (2016) as described below:

| E _adjusted_ = 1/2 * (E + E^T^)  where, E = contact matrix  E^T^ = transposed matrix of E  E adjusted = transformed contact matrix |
| --- |

3. Calibration of the multiplies for each of the aggregated age-group-specific rates

As the age group classification system shown in Table A3-1 requires creation of a high number of model parameters for contact rates, even after the transformation of the matrix for symmetry, we newly defined three aggregate age groups (0y, 1-9y, and 10y+), in line with the process of setting up calibration target (see also Appendix 7), for the purpose of calibration of model parameters. This led to a total of six contact rate between age groups. We defined a multiplier for each of the between-group contact rates to identify the best-fitting rates by varying the multipliers. The final set of the values of the multipliers determined are shown in Table A3-2.

Table A3-2. Calibrated values of the contact rate between age groups

| Age group | 0y | 1y-9y | 10y+ |
| --- | --- | --- | --- |
| 0y | 0.652 | 1.992 | 1.769 |
| 1y-9y | 1.992 | 1.262 | 1.138 |
| 10y+ | 1.769 | 1.138 | 1.262 |

4. Transmission probability per contact

The values of the transmission probabilities per contact were estimated using the following steps: 1) age-group specific transmission probabilities per contact were obtained from a study in England and Wales (see Table A3-3); 2) a multiplier was applied to age-group specific values for age group(s) that belong to each of the three aggregated age groups (0y, 1-9y, 10y+); and 3) finally, the values of the three multipliers were determined as best-fitting values through calibration (see Table A3-4).

Table A3-3. Transmission probability per contact by age group in 1956 for England and Wales (Ref. Choi et al. 2016)

| Age groups | 0y | 1-2y | 3-4y | 5-9y | 10-14y | 1 5-24y | 25-39y | 40-59y | 60+y |
| --- | --- | --- | --- | --- | --- | --- | --- | --- | --- |
| Transmission probability per contact | 8.0  x10^-4^ | 9.0  x10-^4^ | 8.2  x10-^4^ | 8.6  x10-^4^ | 4.4  x10-^4^ | 1.6  x10-^4^ | 1.4  x10-^4^ | 0.05  x10-^4^ | 1.0  x10-^4^ |

Table A3-4. Calibrated values of the multipliers for transmission probability per contact

| Age group | Values of age-group specific multiplier determined |
| --- | --- |
| 0y | 221.607 |
| 1y-9y | 1.423 |
| 10y+ | 1.188 |

**Appendix 4. Infant vaccine coverage**

National data on Infant pertussis vaccine coverage were drawn from two surveys conducted in Brazil, one in 1996 and one in 2007. The only data available for the three states came from the 2007 survey for the capitals of those states: Salvador for Bahia; Curitiba for Parana; Sao Paulo for Sao Paulo.

The National Health and Demography Survey, 1996 (*Pesquisa Nacional Sobre Demografia e Saúde 1996. Sociedade Civil Bem-Estar Familiar no Brasil, BEMFAM, Programa de Pesquisas de Demografia e Saúde (DHS) Macro International Inc. March, 1997. Available at <https://dhsprogram.com/pubs/pdf/fr77/fr77.pdf>) is based on a representative sample of all 5 macro-regions in Brazil (South, Center-West, Northeast, Southeast, and South) and two major States (Rio de Janeiro and São Paulo). In the 1996 survey, the vaccination experience of 2908 children born since January 1991 and alive at survey (3,001 aged <2 years) was documented through home interviews conducted from March 1^st^ through June 30^th^. Vaccine coverage was estimated based on the mother’s report and doses registered in the child’s immunization booklet.

The National Survey of vaccine coverage in urban state capitals in Brazil, 2007 (Barata et al 2012. Journal of Epidemiology and Community Health. 2012;66(10):934-941) is based on a representative sample of 26 state capitals and the Brasilia federal district, which account for 23.7% of total population. Between August 2007 and June 2008, the vaccination experience of 17,749 children less than 18 months of age was documents. Vaccine coverage was estimated based on the mother’s report and doses registered in the child’s immunization booklet (available for 17,295 children).

For both surveys (1996 and 2007) we considered applied doses, irrespective of the timing of the first dose and time interval between doses. Using individual data on applied vaccine doses by children from these two surveys, Colin Sanderson provided us with vaccination coverage modeled by dose and week of age up to 3 years of age for 1996 and 2007. Coverage for each dose was cumulative so that the proportion of infants vaccinated rose monotonically with week of age. The methods are described in Clark A, Sanderson C. Timing of children’s vaccinations in 45 low-income and middle-income countries: an analysis of survey data. Lancet 2009; 373:1543–9 and the 1996 data were prepared for that study. Sanderson applied the same methods to the 2007 survey data, provided by the study principal investigator Dr José Cassio de Moraes. Vaccination coverage for the years between 1996 and 2007 was set by linear interpolation.

From Sanderson’s modeled data we calculated the probability that an infant in each of six age intervals (0-1, 2-3, 4-5, 6-8, 9-11, and 12-23 months) received a given dose. To represent not just coverage but protection the calculations used data for the midpoint of each age interval since a few weeks must elapse after a dose is given before the infant has developed immunity. The calculation of the probabilities is explained in general in the paragraphs below and for each probability in the tables that follow.

For the first dose, the numerator of the probability was the proportion of infants who received a first dose between the midpoint of the previous age interval and the midpoint of the age interval of interest. For example, for the age interval 4-5 months the numerator was the proportion of infants who received the first dose at the midpoint of the 4-5-month age interval, minus those who had received it at the midpoint of the 2-3-month age interval. The denominator was the proportion of infants who had not received a first dose as of the midpoint of the previous age interval. The denominator for the example was thus 1 minus the proportion of infants who had received the first dose at the midpoint of the 2-3-month age interval.

For doses 2 and 3 the numerator of the probability was the proportion of infants who received that dose between the midpoint of the previous age interval and the midpoint of the age interval of interest. The denominator was defined to take into account that the infant must have received dose 1 to be eligible for dose 2, or dose 2 to be eligible for dose 3, and, following the Brazilian schedule for infant vaccination, must have received that dose 2 months or more before the dose of interest. For the age intervals 2-3 months and 4-5 months, each exactly two months long, the denominator was thus the proportion of infants who had received the required dose, say dose 1, at the midpoint of the previous age interval minus those who had received the dose of interest, say dose 2, at the same time point. For the age intervals 6-8, 9-11, and 12-23 months, each longer than two months, the denominators were defined as the proportion of infants who had received the preceding dose two months before the midpoint of the age interval of interest. For example, the denominator for the probability that infants received dose 2 at 18 months was the proportion of infants who had received dose 1 by 16 months, two months earlier, minus the proportion who had received dose 1 at the midpoint of the previous age interval, 9-11 months, or, more specifically, the proportion who had received dose 1 by 69 weeks minus the proportion who had received dose 2 at 45 weeks.

To obtain standard errors for the probabilities, we used the formula: SQRT(p*(1-p))/N) where, p=proportion who received a given dose in a given age interval and N=number of infants in the survey.

Table A4-1. Probability of vaccination by dose and age in 2007

| Dose | Age (month) | Probability | Calculation |
| --- | --- | --- | --- |
| DTP1 | 2-3 | 0.316770 | infants w DPT1 by 12 wks |
| DTP1 | 4-5 | 0.273029 | infants w DPT1 by 21 wks minus 12 wks/ 1 minus DPT1 by 12 wks |
| DTP1 | 6-8 | 0.376524 | infants w DPT1 by 32 wks minus 21 wks/ 1 minus DPT1 by 21 wks |
| DTP1 | 9-11 | 0.514348 | infants w DPT1 by 45 wks minus 32 wks/ 1 minus DPT1 by 32 wks |
| DTP1 | 12-23 | 0.652238 | infants w DPT1 by 78 wks minus 45 wks/ 1 minus DPT1 by 45 wks |
| DTP2 | 4-5 | 0.715383 | infants w DPT2 by 21 wks/infants w DPT1 by 12 wks (2m earlier) |
| DTP2 | 6-8 | 0.765243 | infants w DPT2 by 32 wks minus by 21 wks/infants w DPT1 by 21 wks minus DPT2 by 21 wks |
| DTP2 | 9-11 | 0.834359 | infants w DPT2 by 45 wks minus by 32/infants w DPT1 by 36 wks minus DPT2 by 32 wks |
| DTP2 | 12-23 | 0.937609 | infants w DPT2 by 78 wks minus by 45/infants with DPT1 by 69 wks minus DPT2 by 45 wks |
| DTP3 | 6-8 | 0.743789 | infants w DPT3 by 32 wks/infants w DPT2 by 21 wks |
| DTP3 | 9-11 | 0.734211 | infants w DPT3 by 45 wks minus by 32 wks/infants w DPT2 by 36 wks minus DPT3 by 32 wks |
| DTP3 | 12-23 | 0.914150 | infants w DPT3 by 78 wks minus by 45 wks/infants w DPT2 by 69 wks minus DPT3 by 45 wks |

Table A4-2. Probability of vaccination by dose and age in 1996

| Dose | Age (month) | Probability | Calculation |
| --- | --- | --- | --- |
| DTP1 | 2-3 | 0.699588 | infants w DPT1 by 12 wks |
| DTP1 | 4-5 | 0.563578 | infants w DPT1 by 21 wks minus 12 wks/ 1 minus DPT1 by 12 wks |
| DTP1 | 6-8 | 0.298626 | infants w DPT1 by 32 wks minus 21 wks/ 1 minus DPT1 by 21 wks |
| DTP1 | 9-11 | 0.220444 | infants w DPT1 by 45 wks minus 32 wks/ 1 minus DPT1 by 32 wks |
| DTP1 | 12-23 | 0.263642 | infants w DPT1 by 78 wks minus 45 wks/ 1 minus DPT1 by 45 wks |
| DTP2 | 4-5 | 0.810366 | infants w DPT2 by 21 wks/infants w DPT1 by 12 wks (2m earlier) |
| DTP2 | 6-8 | 0.778305 | infants w DPT2 by 32 wks minus by 21 wks/infants w DPT1 by 21 wks minus DPT2 by 21 wks |
| DTP2 | 9-11 | 0.529659 | infants w DPT2 by 45 wks minus by 32/infants w DPT1 by 36 wks minus DPT2 by 32 wks |
| DTP2 | 12-23 | 0.586053 | infants w DPT2 by 78 wks minus by 45/infants with DPT1 by 69 wks minus DPT2 by 45 wks |
| DTP3 | 6-8 | 0.950715 | infants w DPT3 by 32 wks/infants w DPT2 by 21 wks |
| DTP3 | 9-11 | 0.611181 | infants w DPT3 by 45 wks minus by 32 wks/infants w DPT2 by 36 wks minus DPT3 by 32 wks |
| DTP3 | 12-23 | 0.562642 | infants w DPT3 by 78 wks minus by 45 wks/infants w DPT2 by 69 wks minus DPT3 by 45 wks |

**Appendix 5. Vaccine efficacy**

1. Maternal aP vaccine efficacy

The estimate of maternal aP vaccination effectiveness comes from an English study [Amirthalingam 2014], which found that maternal TdaP vaccine effectiveness in infants younger than 3 months was 91% (95% CI: 84 - 95). We assume that maternal immunization protects infants for the first 3-4 months of life.

2. Infant vaccine efficacy

The efficacy of routine infant vaccine by dose was taken from Juretzko et al. [2002], the only study we found that reports efficacy by dose, and is shown as proportions in the table below.

Table A5. The efficacy of routine infant vaccine by dose

| **DTP** | **Vaccine efficacy (VE)** | **Relative risk**  **(RR)** | **Standard deviation for VE and RR** | **Source** |
| --- | --- | --- | --- | --- |
| First dose | 0.680 | 0.320 | 0.09 | (Juretzko 2002) |
| Second dose | 0.918 | 0.082 | 0.03 |  |
| Third dose | 0.998 | 0.002 | 0.003 |  |

Although Juretzko evaluated an aP vaccine and the models assume a wP vaccine, a 2016 systematic review of efficacy of both vaccine types (aP and wP) in children (Fulton 2016) shows that Juretzko’s results closely resemble those for wP vaccines. The review (Fulton 2016) found that 3 or more doses of wP vaccine were 94% effective against pertussis, compared with 84% for 3 or more doses of aP vaccine.

References

1. Amirthalingam G, Andrews N, Campbell H, Ribeiro S, Kara E, Donegan K, Fry NK, Miller E, Ramsay M. Effectiveness of maternal pertussis vaccination in England: An observational study. Lancet 2014; 384: 1521-28. PMID: 25037990
2. Juretzko P, von Kries R, Hermann M, Wirsing von König CH, Weil J, Giani G. Effectiveness of acellular pertussis vaccine assessed by hospital-based active surveillance in Germany. Clin Infect Dis. 2002 Jul 15;35(2):162-7. PMID: 12087522 DOI: 10.1086/341027
3. Fulton TR, Phadke VK, Orenstein WA, Hinman AR, Johnson WD, Omer SB. Protective Effect of Contemporary Pertussis Vaccines: A Systematic Review and Meta-analysis. Clin Infect Dis. 2016 May 1;62(9):1100-1110. PMID: 26908803 PMCID: PMC4826451 DOI: 10.1093/cid/ciw051

**Appendix 6. Cost data**

All costs are reported as 2014 US$. Except for vaccine purchase costs, which are already in dollars for international purchase through the Pan American Health Organization’s Revolving Fund, all costs were estimated in Brazilian Reals (BRL) and converted to 2014 US dollars using the official Brazilian Central Bank Exchange Rate on Dec 31, 2014 (<http://www4.bcb.gov.br/pec/conversao>) when 1 BRL = 0.43 USD.

1. Costs of vaccination

Maternal vaccine price/dose. Brazil began maternal aP immunization in 2014 using a single dose vaccine of multivalent formulation (adolescent/adult TdaP) purchased through the Pan American Health Organization Revolving Fund. Data was obtained from the Pan American Health Organization Revolving Fund 2014 price list (Source: [http://www.**paho**.org](http://www.paho.org)) and attached.

To the base price per dose (USD 8.905), freight and insurance charges (3%) and a service charge (4.25%) were added, bringing the total to USD 9.55. Further adding an additional 5% wastage rate, as recommended by WHO, would bring the cost for each vaccine dose to **USD 10.028.**

Incremental maternal delivery cost/dose. Delivery cost has not been separately estimated for maternal aP immunization. In Brazil, since Td is being replaced by TdaP, it is assumed that there is no incremental cost of delivering TdaP.

Infant vaccine price/dose. We used the 2014 listed dose price for the a single dose pentavalent vaccine (DTP-HepB-Hib) liquid formulation, which is purchased through the Pan American Health Organization Revolving Fund (Source: [http://www.**paho**.org](http://www.paho.org)). To the base price per dose (USD 2.5251), freight and insurance charges (3%) and a service charge (4.25%) were added, totaling USD 2.708. Further adding an additional 5% wastage rate, as recommended by WHO, would bring the cost for each vaccine dose to **USD 2.84**.

Vaccine delivery cost/dose (for any age group). The cost of delivering a dose of vaccine for any individual was obtained from a primary study conducted in 2013 in Brazil which estimated the cost of the immunization program in the country (Toscano et al. manuscript submitted to Vaccine, 2018).

1. Costs of illness

- Costs for pertussis outpatients

The cost of outpatient management of pertussis was estimated based on standardized guidelines for pertussis case management from the Brazilian Ministry of Health and Tertiary care Reference Hospitals in the country (1)(2)(3). These cost estimates apply to patients whose pertussis does not require hospital care. Costs for hospitalized patients were estimated separately; see below.

Direct medical costs were estimated considering the following cost components, which are the healthcare resources used for the outpatient management of a pertussis patient: Diagnostic exams, medical visits, and medications.

Diagnostic exams included specific pertussis diagnosis (bacterial isolation, considered the gold standard for diagnosis), and complementary exams (chest X-ray for children < 5 years of age and blood cell count for all patients). Two medical visits were assumed for each case. Medications included antipyretic (paracetamol 200mg/mL, 15 mL) and Azithromycin as the first-line antibiotic, as recommended by the guidelines.

For diagnosis and medical visits, unit costs were obtained from the standardized national pricing lists of the Brazilian Public Healthcare System (Sistema de Gerenciamento da Tabela de Procedimentos, Medicamentos e OPM do SUS -(SIGTAP/DATASUS), as depicted in Table 1 below (4). Unit costs were then multiplied by quantities to obtain the estimated cost per case of a patient without complications, managed as an outpatient.

Table A6-1. Unit costs for pertussis diagnosis and management, 2014 (in Brazilian Reais, R$)

| **Items** | **Code in SIGTAP** | **Unit cost in BRL** | **Total costs** |
| --- | --- | --- | --- |
| Thorax X-ray (AP and lateral) | 02.04.03.015-3 | R$ 9.50 | R$ 9.50 |
| Blood cell count | 02.02.02.039-8 | R$ 2.73 | R$ 2.73 |
| Cultures for pertussis identification | 02.02.08.008-0 | R$ 5.62 | R$ 5.62 |
| medical visit | 03.01.01.007-2 | R$ 10.00 | R$ 20.00 |

| **Sub-total costs** | Children < 5 years | R$37.85 |
| --- | --- | --- |
|  | Children 5 years and older | R$ 28.35 |

Source: SIGTAP 2017

For medications, the weighted average of government purchases of a given medication in the 12 months January/2014 - December/2014, according to the National Price Bank (Banco de Preços em Saúde) (5), were used. In the base case we considered Azithromycin as the first-line antimicrobial agent, and in sensitivity analysis, we considered Clarithromycin (which is the alternative recommended antimicrobial agent with higher costs). In addition, paracetamol use for 4 days was considered in the following dosage: 1) <8 years, 1 drop/kg; 3 times/day, 2) 8 years and older 40 drops; 3 times/day. Azithromycin was considered in both powder for oral suspension (600mg) for children <13 years old, or 250mg or 500mg capsules depending on recommended dose by weight. The following age groups were considered: 0-1m, 2-3m, 4-5m, 6-8m, 9-11m, 12-23m, 2-4 years, 5-9 years, 10-14 years, 15-19 years, and 20 years and older (adults). Average body weights for each age group by gender were estimated based on standardized WHO Z-scores curves for weight and height (6). Total cost by cost component and by age group is presented below in Table 2.

Table A6-2. Costs for outpatient pertussis case management, by age group, in Brazilian Reais (R$)*

|  |  |  |  |  | **Sensitivity Analysis, considering second line antibiotic** | |
| --- | --- | --- | --- | --- | --- | --- |
| **Age group** | **Diagnosis** | **Medical visit** | **Medications** | **Total Cost** | **Medications** | **Total Cost** |
| < 3 years | R$18.15 | R$20.00 | R$2.21 + R$0.54 | R$40.90 | R$ 26.50+R$0.54 | R$64.99 |
| 3-4 years | R$18.15 | R$20.00 | R$2.21 + R$0.54 | R$40.90 | R$ 52.60+R$0.54 | R$91.29 |
| 5 years | R$8.35 | R$20.00 | R$2.21 +R$0.54 | R$31.10 | R$ 52.60+R$0.54 | R$81.49 |
| 6 years | R$8.35 | R$20.00 | R$2.21 +R$0.54 | R$31.10 | R$ 52.60+R$0.54 | R$81.49 |
| 7 years | R$8.35 | R$20.00 | R$2.21 +R$0.54 | R$31.10 | R$ 52.60+R$0.54 | R$81.49 |
| 8 years | R$8.35 | R$20.00 | R$2.21 +R$0.54x2 | R$31.64 | R$ 52.60+R$0.54x2 | R$82.03 |
| 9 years | R$8.35 | R$20.00 | R$2.21 +R$0.54x2 | R$31.64 | R$ 52.60+R$0.54x2 | R$82.03 |
| 10-11 years | R$8.35 | R$20.00 | R$2.21 +R$0.54x2 | R$31.64 | R$ 76.64+R$0.54x2 | R$106.07 |
| 10-12 years | R$8.35 | R$20.00 | R$4.42 +R$0.54x2 | R$33.85 | R$ 76.64+R$0.54x2 | R$106.07 |
| 13 years | R$8.35 | R$20.00 | R$6.63 +R$0.54x2 | R$36.06 | R$ 76.64+R$0.54x2 | R$106.07 |
| 14 years | R$8.35 | R$20.00 | R$10.66+R$0.54x2 | R$40.09 | R$76.64+R$0.54x2 | R$106.07 |
| 15-19 years | R$8.35 | R$20.00 | R$10.66+R$0.54x2 | R$40.09 | R$76.64+R$0.54x2 | R$106.07 |
| Adults | R$8.35 | R$20.00 | R$10.66+R$0.54x2 | R$40.09 | R$50.32+R$0.54x2 | R$79.75 |

* Costs estimated in 2014 in Brazilian Reais converted to US dollars considering the 2014 official exchange rate where R$1.00=0.43USD in 2014

- Costs for hospitalized pertussis patients, by survived or died

The average costs of inpatient treatment for pertussis cases, stratified by age sub-group and by patient outcome (alive at the end of hospitalization or died during hospitalization), and their standard errors, were obtained from reimbursements paid for all pertussis cases hospitalized in 2014 in the hospitals of the Brazilian National Public Health system (SUS), which covers 75% of the Brazilian population.

Reimbursements include direct medical and non-medical costs. Direct medical costs include hospital stay, healthcare professional services, and physical therapy. Non-medical costs include the stay of a parent or caregiver accompanying the hospitalized child, which is paid for by SUS to the hospital.

Reimbursements for each cost item are standardized nationwide within SUS (SIGTAP) (http://sigtap.datasus.gov.br/tabela-unificada/app/sec/procedimento/exibir/0303010037/02/2014). The hospital stay is valued based on a standard stay by ICD10 diagnostic code (A37 for pertussis). Pertussis reimbursement is BRL 793.69 for a hospital stay of up to 12 days, after which an additional BRL 20.00 per day is paid. The standard reimbursement for healthcare professional services for pertussis is BRL 72.22, which may increase depending on the need for additional specialty professionals. Each physical therapy session is an additional BRL 6.35. Each day of hospital stay of an accompanying parent or caregiver is reimbursed at BRL 8.00.

Total hospitalization costs by age group is presented below in Table 3. Costs are further stratified by patient outcome (dead or alive at the end of hospitalization). Number of cases in each strata, average costs, standard error, standard deviation, and minimum and maximum observed reimbursement values are also reported for each strata. Highlighted in yellow are strata for which no cases and thus no reimbursement values were obtained. To generate estimates for these strata, data from the other age groups where interpolated linearly

.

Table A6-3. Costs for hospitalized pertussis case, by outcome and age group, in Brazilian Reais (R$)*

| **Age group and outcome** | | **Mean (R$)** | **N** | **Std. Deviation** | **Std. Error of Mean** | **Minimum** | **Maximum** |
| --- | --- | --- | --- | --- | --- | --- | --- |
| 0-1m | Alive | 1644,09 | 1554 | 2509,22 | 63,65 | 47,27 | 29420,27 |
|  | Died | 2647,04 | 30 | 2391,44 | 436,61 | 865,91 | 11788,61 |
|  | Total | 1663,08 | 1584 | 2510,04 | 63,07 | 47,27 | 29420,27 |
| 2-3m | Alive | 1264,50 | 1672 | 1839,51 | 44,99 | 44,22 | 29576,04 |
|  | Died | 3362,82 | 14 | 3386,80 | 905,16 | 881,91 | 13310,06 |
|  | Total | 1281,93 | 1686 | 1865,60 | 45,43 | 44,22 | 29576,04 |
| 4-5m | Alive | 1094,51 | 677 | 1006,60 | 38,69 | 47,27 | 15517,05 |
|  | Died | 2365,92 | 3 | 1416,03 | 817,55 | 960,40 | 3792,23 |
|  | Total | 1100,12 | 680 | 1010,83 | 38,76 | 47,27 | 15517,05 |
| 6-8m | Alive | 1144,54 | 286 | 1622,76 | 95,96 | 47,27 | 21291,11 |
|  | Total | 1144,54 | 286 | 1622,76 | 95,96 | 47,27 | 21291,11 |
| 9-11m | Alive | 952,20 | 108 | 314,09 | 30,22 | 47,27 | 1947,67 |
|  | Died | 15840,58 | 1 | NA | NA | 15840,58 | 15840,58 |
|  | Total | 1088,79 | 109 | 1459,92 | 139,83 | 47,27 | 15840,58 |
| 12-23m | Alive | 1141,72 | 146 | 1715,83 | 142,00 | 47,27 | 19478,43 |
|  | Died | 1207,62 | 3 | 421,33 | 243,25 | 873,91 | 1681,06 |
|  | Total | 1143,05 | 149 | 1699,08 | 139,19 | 47,27 | 19478,43 |
| 2-4a | Alive | 961,13 | 208 | 593,24 | 41,13 | 47,27 | 6448,15 |
|  | Died | 3738,22 | 1 | NA | NA | 3738,22 | 3738,22 |
|  | Total | 974,42 | 209 | 622,21 | 43,04 | 47,27 | 6448,15 |
| 5-9a | Alive | 944,77 | 122 | 291,42 | 26,38 | 47,27 | 3392,86 |
|  | Total | 944,77 | 122 | 291,42 | 26,38 | 47,27 | 3392,86 |
| 10-17a | Alive | 913,85 | 46 | 244,60 | 36,06 | 47,27 | 1845,98 |
|  | Died | 6622,73 | 1 | NA | NA | 6622,73 | 6622,73 |
|  | Total | 1035,31 | 47 | 867,16 | 126,49 | 47,27 | 6622,73 |
| 18-39a | Alive | 913,79 | 25 | 124,77 | 24,95 | 865,91 | 1428,98 |
|  | Total | 913,79 | 25 | 124,77 | 24,95 | 865,91 | 1428,98 |
| 40-64a | Alive | 1049,89 | 13 | 278,63 | 77,28 | 865,91 | 1612,12 |
|  | Died | 1797,70 | 4 | 1863,58 | 931,79 | 865,91 | 4593,07 |
|  | Total | 1225,85 | 17 | 903,50 | 219,13 | 865,91 | 4593,07 |
| 65+ys | Alive | 2026,42 | 13 | 3592,75 | 996,45 | 52,22 | 13813,63 |
|  | Died | 1382,90 | 4 | 870,62 | 435,31 | 869,91 | 2685,81 |
|  | Total | 1875,00 | 17 | 3146,77 | 763,20 | 52,22 | 13813,63 |
| Total | Alive | 1319,72 | 4870 | 1914,22 | 27,43 | 44,22 | 29576,04 |
|  | Died | 2887,46 | 61 | 3006,85 | 384,99 | 865,91 | 15840,58 |
|  | Total | 1339,12 | 4931 | 1938,81 | 27,61 | 44,22 | 29576,04 |

REFERENCES

1. Ministério da Saúde. Guia de Vigilância Epidemiológica [Internet]. Série A. Normas e Manuais Técnicos. 2009. 819 p. Available from: http://bvsms.saude.gov.br/bvs/publicacoes/guia_vigilancia_epidemiologica_7ed.pdf

2. BRASIL. Ministério da Saúde. Guia de Vigilância em Saúde. v1 ed. Ministério da Saúde, editor. Brasília: Secretaria de Vigilância em Saúde. Coordenação-Geral de Desenvolvimento da Epidemiologia em Serviços.; 2017.

3. Pediatria SB de. Coqueluche:recomendações atuais. 2013. p. http://www.sbp.com.br/imprensa/detalhe/nid/coquelu.

4. BRASIL. Ministério da Saúde. Sistema de Gerenciamento da Tabela de Procedimentos, Medicamentos e OPM do SUS [Internet]. 2014. Available from: http://sigtap.datasus.gov.br/tabela-unificada/app/sec/inicio.jsp

5. BRASIL. Banco de preços em saúde. 2014. Available from: http://portalms.saude.gov.br/gestao-do-sus/economia-da-saude/banco-de-precos-em-saude

6. WHO WHO. The WHO Child Growth Standards [Internet]. 2007 [cited 2017 Oct 10]. Available from: http://www.who.int/childgrowth/standards/weight_for_age/en/

**Appendix 7. Calibration methods**

1. Calibration target

For a pertussis dynamic model, in comparing model projected outcomes with empirical data, it is important to check age (group)-distribution of the modeled outcomes as well as the total number of outcomes against empirical data. In Brazil, approximately 50-60% of notified (i.e., actually reported) cases are reported among infants (<1y), and, among the rest of the notified cases reported in individuals >1y, about 50% of cases are reported from 1-9y age group. Also, it is reported that there are considerable differences in biological characteristics and social behavior between the two age groups 1-9y and 10y+. In this light, in choosing a calibration target, we decided to use age group-stratified notification cases (or incident cases when adjusting for under-reporting) for three age groups: <1y, 1-9y, and 10y+.


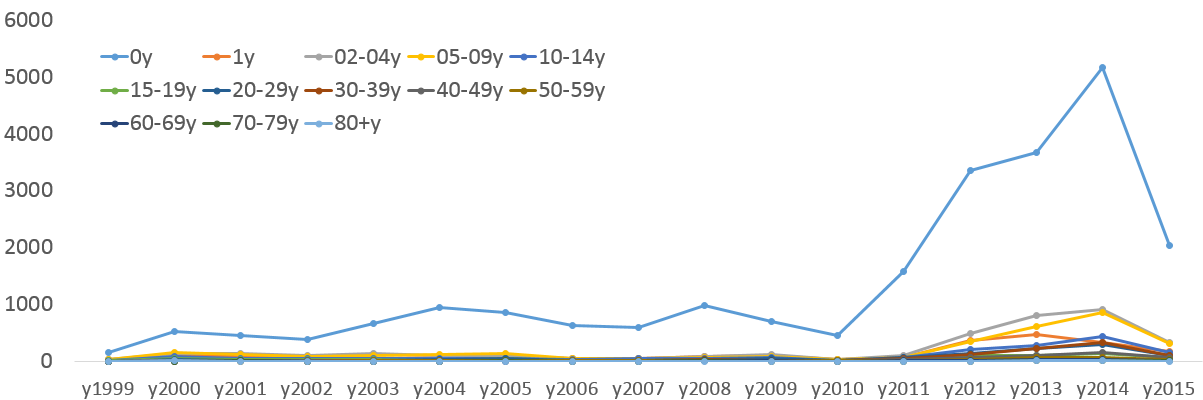


Figure A7-1. Number of notified (actually reported) by age group (all age group) from 1999 to 2015


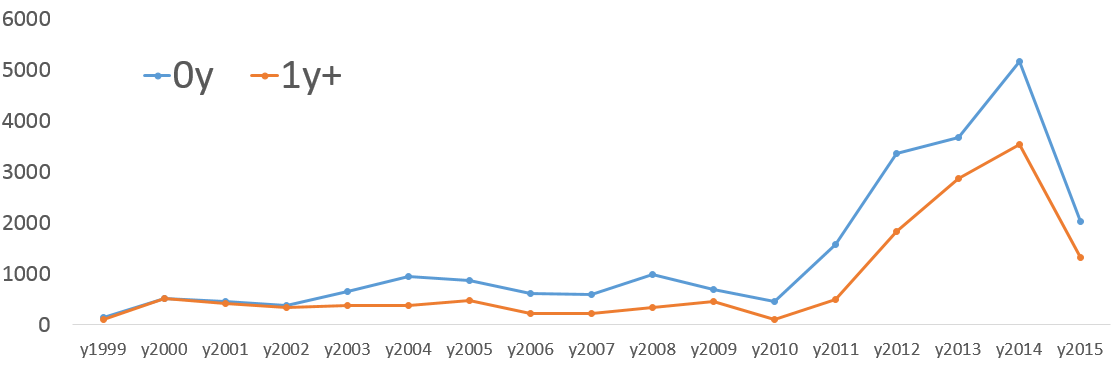


Figure A7-2. Number of notified (actually reported) cases by age group (0y and 1y+) from 1999 to 2015


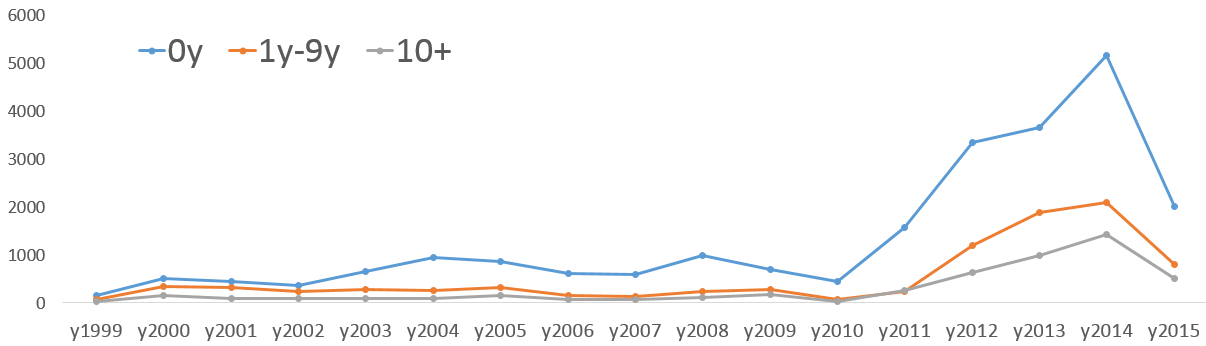


Figure A7-3. Number of notified (actually reported) by age group (0y, 1-9y, 10y+) from 1999 to 2015

2. Goodness-of-fit (GOF) measurements

Since our model is employing multi-targets (3 age group-stratified notifications), we need to quantify GOF for each target. Between the two common quantitative measures of GOF, AIC (likelihood based approach) and SSE, we chose to use AIC as we considered AIC would be more appropriate for the partially observed Markov process model, which incorporates a measurement model based on Poisson distribution. AIC for each parameter set can be calculated as below:


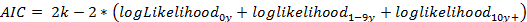

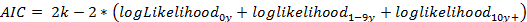

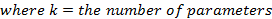

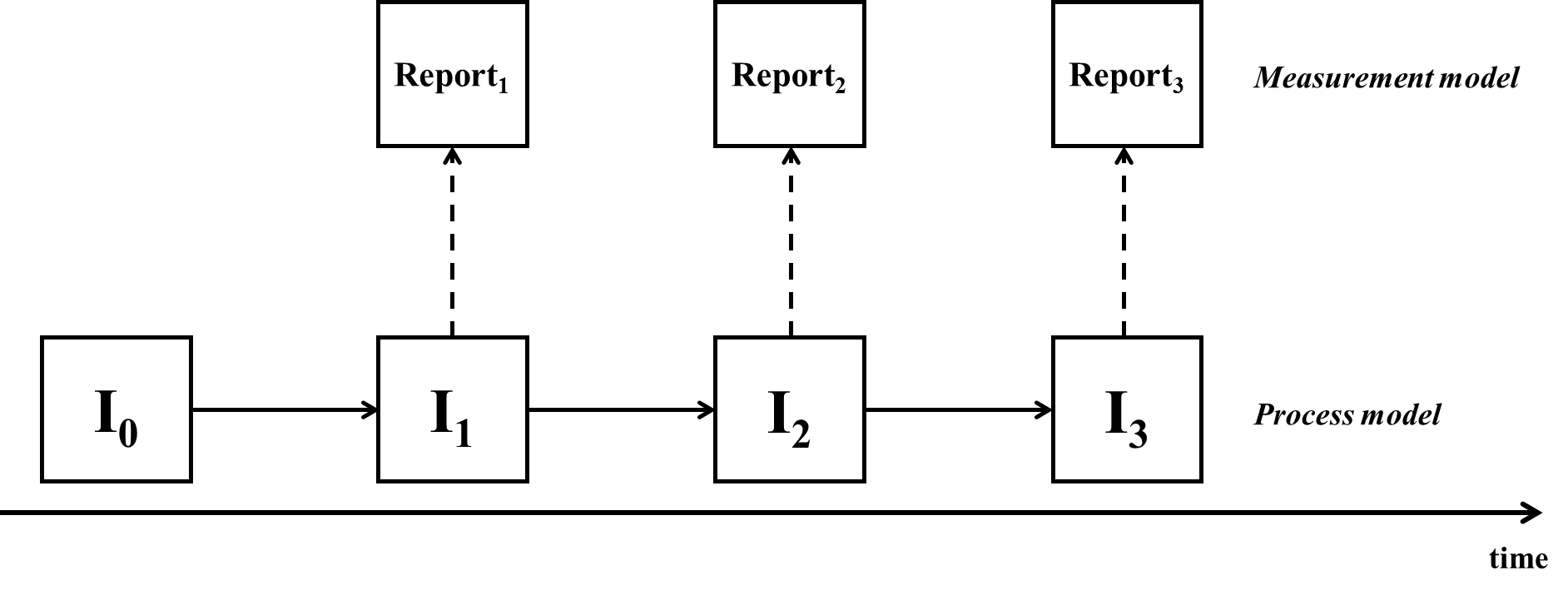


Figure A7-4. Partially observed Markov Process Model

Note: While the process model has developed with deterministic approach, the measurement model suppose stochastic approach with Poisson distribution

3. Parameter searching method

We used a two-step method incorporating two parameter searching methods to find optimal parameter sets yielding the best model outputs that are well-fitted to the reported data

Step 1. Acceptable window approach

Considering that 13 parameters to be varied (including reporting rates by age groups, contact matrix multiplier, multipliers for transmission probabilities by age groups, and waning rate of vaccine-induced immunity) are substantially sensitive and relatively uncertain and the entire calibration process is highly computationally burdensome, we conducted preliminary sensitivity analysis to identify parameter sets that produce results in the acceptable ranges, as shown in Figure A7-5.

**Grid searching & acceptiable window approach**

**Disease incidence**

**time**

**Observed data**

**& acceptiable window**

**unaccepted parameters**

**accepted parameters**


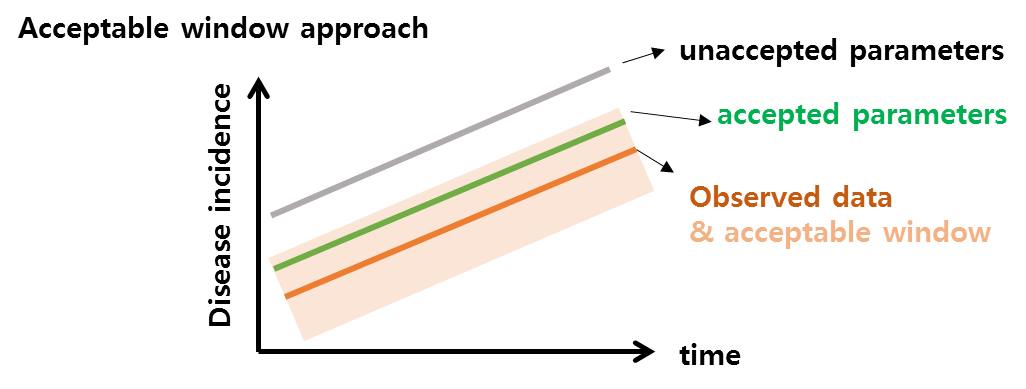


Figure A7-5. Acceptable window approach

Step 2. GOF estimation with Latin hypercube sampling

While there are some algorithms (such as iterative filtering and Nelder-Mead algorithm) that optimize objective function (i.e., likelihood in our model), they are developed for single-objective optimization. However, as describe before, our model has a three targets, and thus cannot employ such algorithms for single-objective optimization. Instead, we determined to generate 100,000 parameter sets for each model by Latin hypercube sampling method based on the parameter sets identified in Step 1. (Lower and upper bounds for the Latin hypercube sampling were determined as 90% and 110% of the parameter ranges identified from the previous step.) Likelihood of the simulated results from the generated parameters were calculated, and this enabled estimation of AIC. The rule for selecting a best-fitting model was to identify a model with the lowest AIC value.

4. Model selection

Using the two step method, 100,000 simulation results with AIC values were produced for each variant of the dynamic model. Model 4 was originally considered a candidate model but was excluded from the candidate models during the model parameterization stage since it was realized that some features of the model structure are hypothetical and biologically less plausible and thus have a potential to yield higher reinfection than primary infection under certain parameter values. Based on the simulation results, among the remaining three models (Models 1 to 3) Model 3 was identified to yield the lowest AIC and thus was selected as the bust-fitting model, as shown in the graphs below (The left side box plot shows the entire ranges of AIC values for Models 1 to 3; The right side graph magnifies the bottom part of the left side box plot for ease of discernment of the lowest AIC values for each Model).


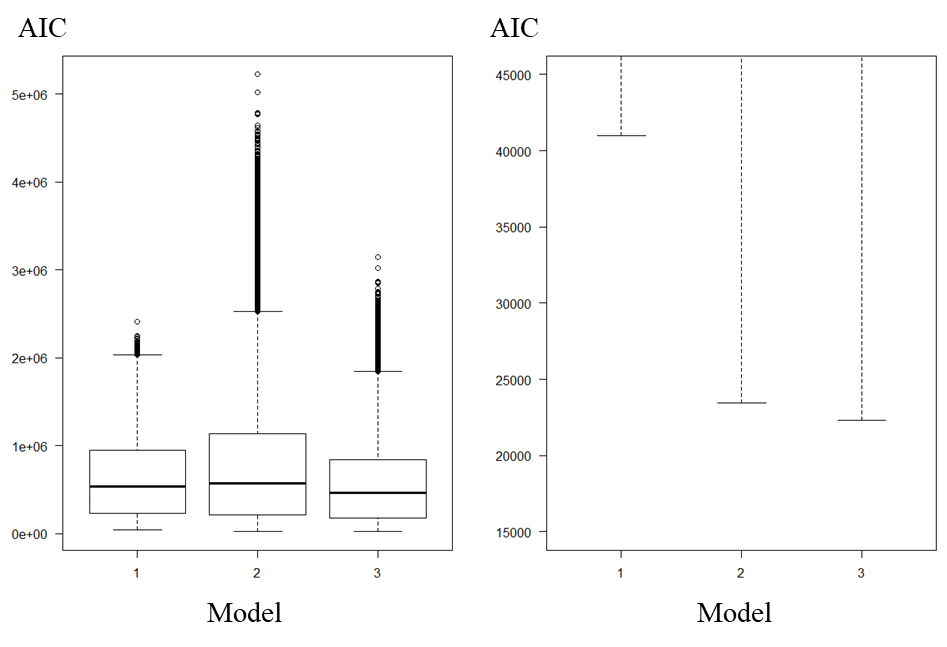


Figure A7-8. Selecting the best model using a likelihood-based goodness-of-fit: Distributions of AIC scores by different model structure

**Appendix 8. CHEERS Checklist completed**


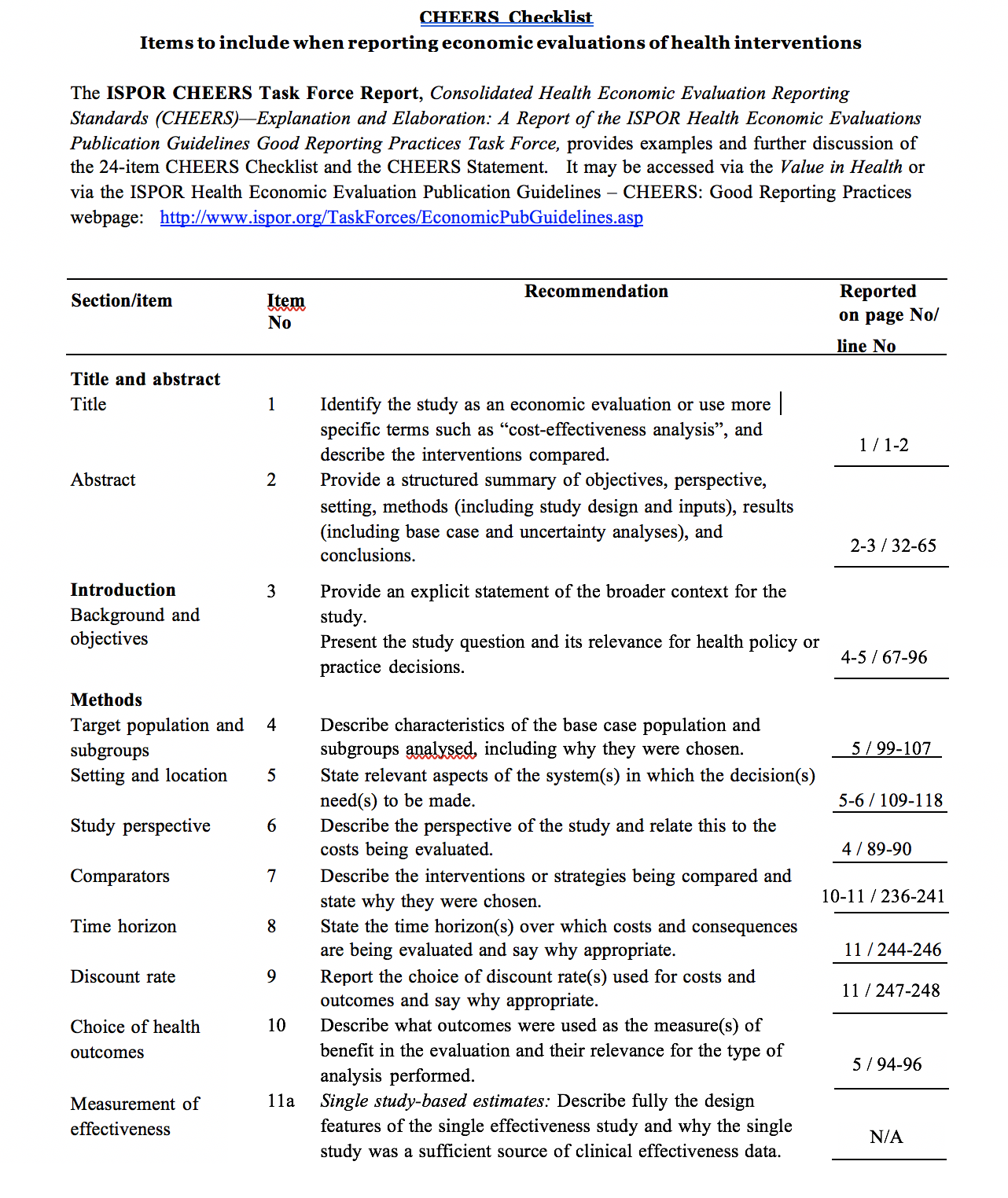


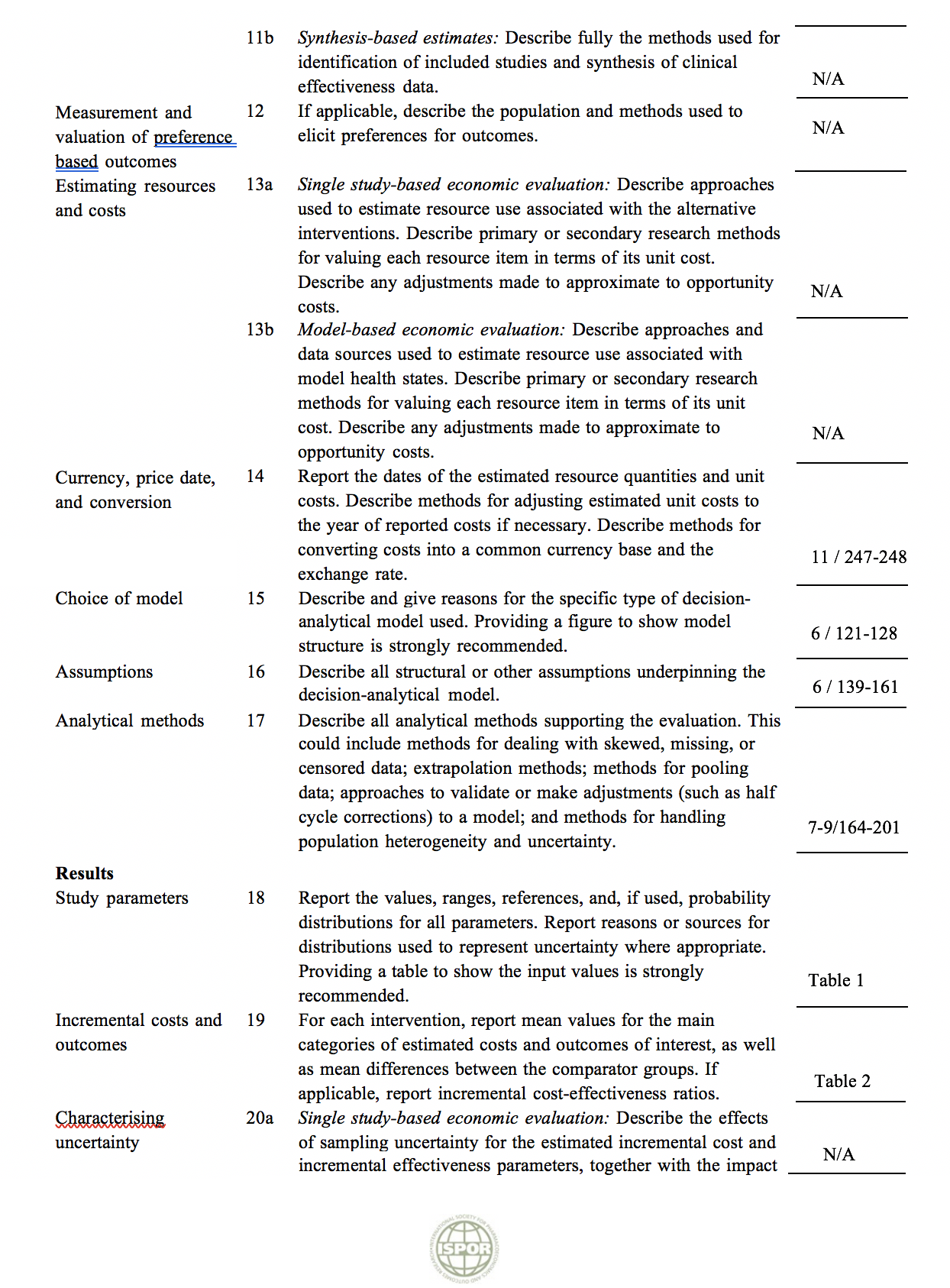


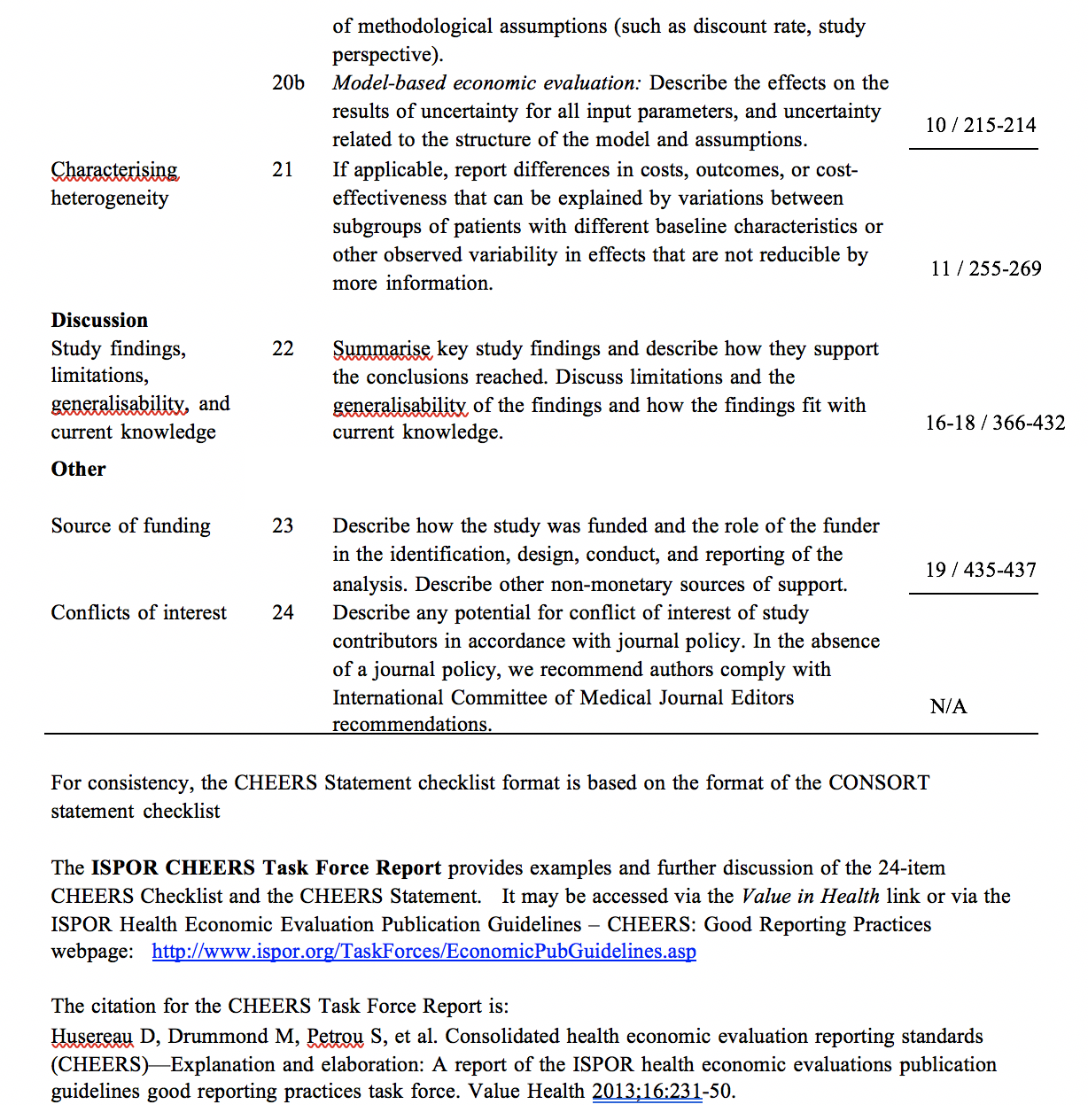

Supplement: Supplementary data 1 [file mmc1.docx]
